# The Splicing Factor PTBP1 Represses *TP63 γ* Isoform Production in Squamous Cell Carcinoma

**DOI:** 10.1158/2767-9764.CRC-22-0350

**Published:** 2022-12-20

**Authors:** William Taylor, Stéphane Deschamps, David Reboutier, Luc Paillard, Agnès Méreau, Yann Audic

**Affiliations:** Univ Rennes, CNRS, IGDR (Institut de Genetique et Developpement de Rennes) – UMR 6290, F-35000 Rennes, France.

## Abstract

The *TP63* gene encodes the p63 transcription factor. It is frequently amplified or overexpressed in squamous cell carcinomas. Owing to alternative splicing, p63 has multiple isoforms called α, β, γ, and δ. The regulatory functions of p63 are isoform specific. The α isoform inhibits the epithelial-to-mesenchymal transition (EMT) and controls apoptosis, while the γ isoform promotes EMT. Using The Cancer Genome Atlas data, we observed that a higher proportion of the *TP63γ* isoform is a detrimental factor for the survival of patients with head and neck squamous cell carcinoma (HNSCC) and is accompanied by the downregulation of desmosomal genes. By a correlation-based approach, we investigated the regulation of the production of the *TP63γ* isoform. According to our analysis of GTEx data, the expression of the RNA-binding protein PTBP1 (polypyrimidine tract binding protein 1) is negatively correlated with the abundance of *TP63γ* in several tissues*.* Accordingly, we demonstrated that PTBP1 depletion in HNSCC cell lines, keratinocyte or Xenopus embryos leads to an increase in *TP63γ* isoform abundance. By RNA immunoprecipitation and *in vitro* interaction assays, we showed that PTBP1 directly binds to *TP63* pre-mRNA in close proximity to the *TP63γ*-specific exon. Intronic regions around the *TP63γ*-specific exon were sufficient to elicit a PTBP1-dependent regulation of alternative splicing in a splice reporter minigene assay.

Together, these results identify *TP63γ* as an unfavorable prognostic marker in HNSCC, and identify PTBP1 as the first direct splicing regulator of *TP63γ* production and a potential route toward *TP63* isoform control.

**Significance:**

Quantifying *TP63γ* isoforms in patients’ tumors could allow for the early detection of patients with HNSCC with an early loss in desmosomal gene expression and poor prognostic. The identification of PTBP1 as a transacting factor controlling *TP63γ* production may allow to control *TP63γ* expression.

## Introduction

The *TP63* gene encodes a conserved transcription factor, p63, controlling epithelial development and homeostasis (1, 2). In humans, heterozygous mutations in *TP63* lead to developmental syndromes affecting ectodermal tissue derivatives (3). Overexpression or amplification of *TP63* is observed in multiple cancer types generally from epithelial origin (4) and p63 staining therefore serves as a classification biomarker in some malignancies including skin cancer (5). The staining for p63 enables discrimination between carcinoma and non-carcinoma breast cancer types, and between lung carcinoma and lung adenocarcinoma in combination with other markers (6, 7). *TP63* is also a prognostic marker (8) as its loss is associated with metastatic progression in head and neck squamous cell carcinoma (HNSCC).

**FIGURE 1:**
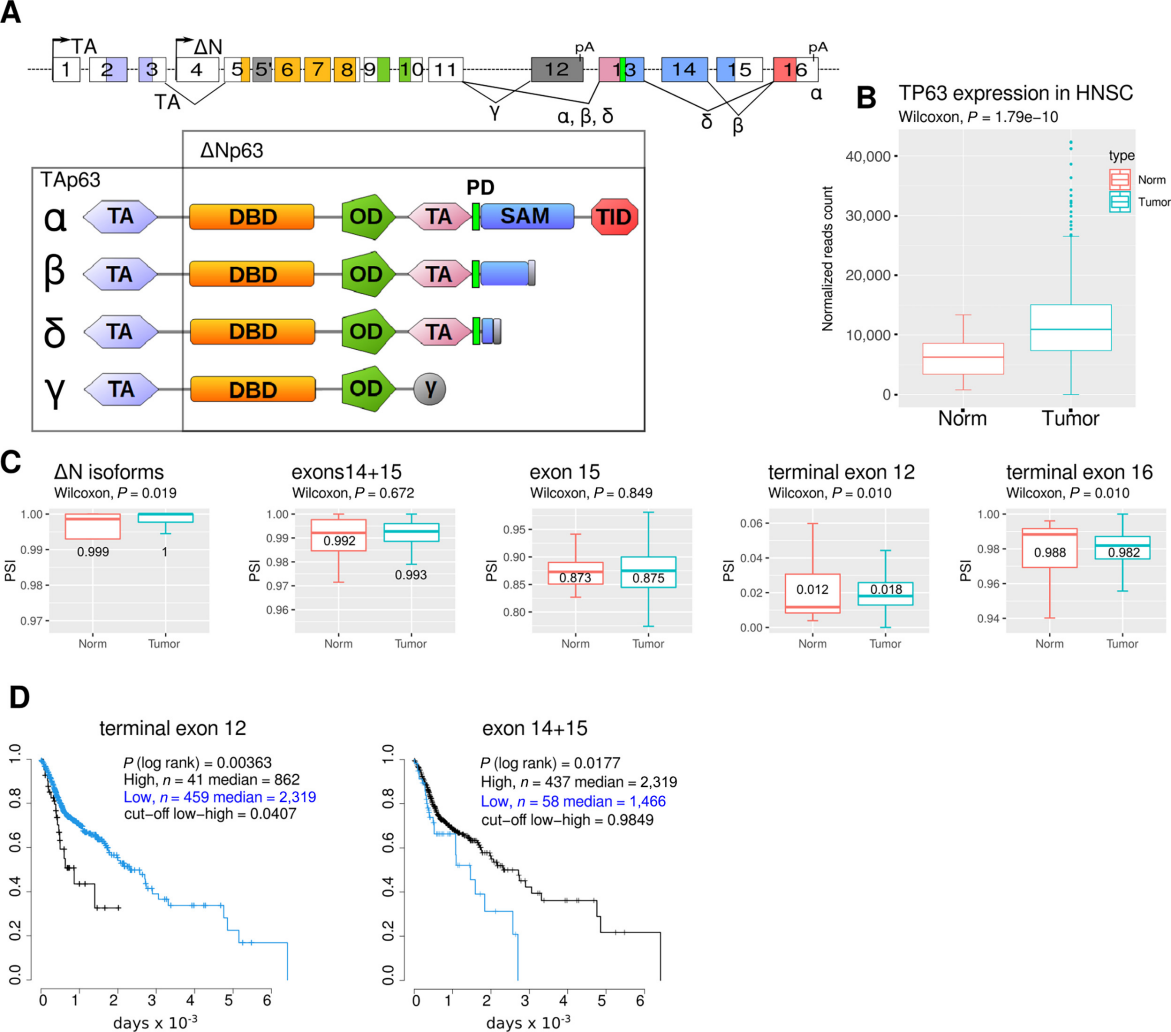
*TP63* splicing from patients with HNSCC. **A,***TP63* exon/intron structure (top diagram) and corresponding encoded protein isoforms with color-encoded peptidic domains. TA, ΔN denote the promoters. Exons are numbered according to GTEX. Alternatively spliced junctions and CPA events are indicated with solid lines joining exons and pA, respectively. Terminal exon 16 is present in isoforms α, β, or δ, and terminal exon 12 in isoform γ. The cassette exons 14 and 15 are present in isoform α while the isoform β lacks exon 15 and the isoform δ lacks both. The exons are colored according to the domain structure. TA, transactivation domain; DBD, DNA-binding domain; OD, oligomerization domain; SAM, sterile alpha motif; TID, transactivation inhibitory domain; PD, phosphodegron. **B,** Genewise expression of *TP63* in normal and tumor samples from patients with HNSCC. **C,** Quantification of *TP63* splicing by measuring the inclusion of specific exons (PSI) in normal and tumor samples. The median PSI is indicated. Differential expression between normal and tumor samples is assessed using Wilcoxon rank-sum test. **D,** Patients were segregated on the basis of the high or low inclusion of exon 12 (threshold = 4.07%) or exon 14+15 (threshold = 98.49%) in tumor samples. Patient survival was assessed on a Kaplan–Meier graph in the two classes and statistical differences appraised by a log-rank test.

The *TP63* gene structure is evolutionarily conserved. Beside exons, several intronic regions also present similarities among vertebrates. The complexity of the *TP63* gene structure, owing to alternative promoters and alternative splicing or polyadenylation, allows for the production of multiple protein isoforms (Fig. 1A). This conserved complexity suggests some evolutionary pressure presumably associated with the function of the different isoforms and underlines the importance of their continued regulation (9, 10).

Two promoters (TA and ΔN) define the major N-terminal variants of p63 (Fig. 1A). The first promoter generates mRNAs encoding TAp63 isoforms comprising the N-terminal transactivation (TA) domain. The mRNAs produced from the second promoter encode only a partial TA domain and the encoded proteins are the ΔNp63 isoforms. The ΔNp63 isoforms are the most abundant ones in epithelial tissues, where they are present mainly in basal cells. They carry most of the p63 functions required for epidermal proliferation and differentiation. The TA isoforms are preferentially expressed in the ovaries during female germline differentiation where they protect the genome from accumulating DNA damage in a way similar to p53 in somatic cells (11).

Several C-terminal ends are produced from combinations of cassette and alternative terminal exons (Fig. 1A). These isoforms encode p63 proteins harboring different combinations of the C-terminal domains. The longest isoform p63α contains a phosphodegron (PD; ref. 12), a sterile-alpha-motif (SAM; ref. 13), a transcriptional inhibitory domain (14), and a regulatory sumoylation region (15). The shortest mRNA isoform *TP63γ* encodes only the oligomerization and DNA-binding domains. *TP63γ* mRNA is generated by the use of an internal terminal exon and thus has a different 3′ untranslated region to the other C-terminal isoforms. Because all p63 isoforms contain the oligomerization domain, they may all interfere with each other's functions. Most described functions of the p63α isoform concern its requirement for epithelial development and homeostasis, whereas the p63γ isoform alone is unable to support proper epithelial development (16). Instead, p63γ promotes the onset of an epithelial-to-mesenchymal (EMT) transition when overexpressed in keratinocytes (17), promotes osteoblastic differentiation (18) and favors terminal myogenesis (19). Metastatic potential is a major parameter of the severity of HNSCC and is enhanced by the acquisition of EMT properties (20). Differential splicing regulation altering the abundance of C-terminal p63 isoforms could therefore promote acquisition of metastatic properties and be detrimental to patients.

The functions of p63 are therefore strongly dependent on the nature of the isoforms and on the regulation of the production of the different 5′ and 3′ isoforms in a tissue-specific manner. Despite the acknowledged importance of the differential role of the p63 C-terminal isoforms, the regulation of the multiple alternative splicing events involved in *TP63* pre-mRNA maturation has remained unaddressed since the cloning of the gene in the late 90s (21).

Regulation of alternative splicing events requires the assembly of the spliceosome machinery onto splice sites [noted 5′ splice site (5′SS) and 3′ splice site (3′SS) with regards to the introns] and/or the definition of the cleavage and polyadenylation sites (CPA; ref. 22). The tissue-specific choices among different alternative splicing and polyadenylation events are generally controlled by a combination of regulatory sequence elements, silencers or enhancers, and their cognate trans-acting factors, generally RNA-binding proteins (RBP). The differential tissue distribution of these RBPs will often account for the tissue-specific regulation of alternative splicing (23). We reasoned that by analyzing relative abundance of RBPs and *TP63* alternative splicing events in RNA-sequencing (RNA-seq) data from many tissues we could prioritize the RBPs most likely to intervene in *TP63* splicing.

Here, by combining exploration of The Cancer Genome Atlas (TCGA) and GTEX data, RNA/protein biochemistry, and reverse genetics experiments, we show that higher inclusion of the γ exon is associated with poorer survival of patients with HNSCC and we identify the RBP PTBP1 as a direct inhibitor of the inclusion of the γ exon in HNSCC cell lines. Furthermore, patients’ primary tumors are characterized by a decreased expression of desmosomal gene that are known actors of tumors suppression (24), suggesting a loss of epithelial characteristics. These results identify *TP63γ* as an unfavorable prognostic marker in HNSCC, and identify PTBP1 as the first direct splicing regulator of *TP63γ* production and a potential route toward p63 isoforms control.

## Materials and Methods

### Cell Lines

The panel of HNSCC cell lines (TCP-1012:A253, FaDU, SCC-25, SCC9) was acquired from ATCC in July 2019 and authenticated by the provider by short tandem repeat profiling. Authenticated HaCaT cells were obtained from Cell Lines Service in September 2020 (CLS GmbH). All cell lines used for the experiments were at least three passages and at most 20 passages from thawing, at which point they were systematically discarded. Cells were not tested for *Mycoplasma* infection. HaCaT, FaDu, HeLa, and Detroit 562 cells were cultured in DMEM (Gibco), A253 cells were cultured in Mc Coy's 5A (Gibco), SCC-9 and SCC-25 cells were cultured in DMEM/F12 (Gibco) supplemented with 400 ng/mL hydroxycortisone (H0888, Sigma-Aldrich). For siRNA depletion, cells were seeded on 6-well plates at 150,000 cells per well. After seeding, a jetPRIME transfection mix (Polyplus) was added, containing 100 pmol of target siRNA or negative controls.

### *Xenopus* Embryos Microinjection

*Xenopus laevis* eggs were obtained from wild-type (WT) females and fertilized using standard procedures and following national regulation under protocol (APAFIS#7109; ref. 25). When indicated, 30 ng of MoPtbp1 (26) or 30 ng of control morpholino (GeneTools) were injected into each blastomere of two-cell embryos in a volume of 13.8 nL, using a Nanoject II (Drummond). For rescue experiments, 1 fmol of mRNA-encoding morpholino-resistant Ptbp1-V5R was coinjected. Embryos were allowed to develop at 22°C and were collected at stage 26 according to Nieuwkoop and Faber stages (27). Total RNA was extracted using RNeasy columns (Qiagen) from pools of three embryos. RNA was analyzed by RT-PCR for 25 cycles using radiolabeled PCR primers.

### Minigene Construction and Analysis

The *TP63* exon 12 minigene and its mutated versions were constructed by Gibson assembly of *β-globin* and *TP63* gene fragments obtained by PCR amplification of HaCaT genomic DNA using oligonucleotides pairs hGbE1_E2, hTP63_gamma, hGb_E3 and cloned into ApaI, NheI digested pmirglo (Promega). Mutations of the 3′SS and/or CPA were conducted using geneblocks (IDT) overlapping the regions to be mutated. Plasmids were controlled by sequencing. HaCaT cells were transfected with minigene plasmid and selected using G418 at 800 μg/mL until naive cell died then maintained in 400 µg/mL (Gibco). Expression and splicing of the minigene was assessed by qRT-PCR. All oligonulceotides are presented in the reagent table.

### RNA Extraction, Reverse Transcription, and qRT-PCR

Total RNA was extracted from cells using nucleospin RNA kits (Macherey Nagel, #740955.50) following the manufacturer's instructions. A total of 1 μg of total RNA was reverse transcribed with superscript II enzyme (Invitrogen) and random primers (Invitrogen #58875). Dilutions of cDNA (1:20) were amplified with PowerSybr Green Master mix (Applied Biosystems, #4367659) using a Quantstudio (TM) 7 flex real-time PCR 384-well system. Two reference genes were used for qRT-PCR, *RPLP0,* and *BTUB*.

### Western Blot Analysis

Cells were washed with 1X PBS and lysed in 2X Laemmli sample buffer (4% SDS, 20% glycerol, 0.125 mol/L Tris-Hcl pH 6.8, 2.5% β-mercaptoethanol, 0.2% bromophenol blue). Denatured protein samples (10′, 95°C) were loaded onto 8%–12% SDS-PAGE gels. Transfer onto nitrocellulose membranes (Amersham Protran 0.45 μm NC) was performed using the trans-blot turbo transfer system (Bio-Rad). Membranes were blocked for 1 hour with a 5% fat-free milk TBST solution [1X TBS (20 mmol/L TRIS, 150 mmol/L NaCl, pH 7.6), 0.1% Tween 20], incubated with primary antibody solutions (16 hours, 4°C). After three washes in TBST (5′), the membranes were incubated with anti-mouse or anti-rabbit secondary antibodies coupled to horseradish peroxidase (1 hour at 20°C), washed three times in TBST (5′). Revelation was performed on an Amersham AI680 imager with either ECL-select (Amersham) or West pico PLUS (Thermo Fisher Scientific) chemiluminescent substrate, depending on anticipated signal strength.

siRNA, oligonucleotides, antibodies, and additional details on reagents and cell lines are listed in Supplementary Table S1.

### Statistical and Data Analyses

*TP63* isoforms and splicing in HNSCC patient samples were quantified using clinical and expression data obtained from firebrowse.org and splicing quantification from TCGA spliceSEQ (28). The percent-spliced-in (PSI) metric is computed as the ratio of normalized reads supporting inclusion of an exon to the total normalized reads for that event. All expression, splicing, and survival analysis were conducted in R. For Kaplan–Meier estimation, the *survival* package was used and a log-rank test (29) applied. Prognostic marker genes were obtained from https://www.proteinatlas.org. Gene Ontology (GO) enrichment analysis was conducted using the *topGO* package.

To identify RBPs with tissue expression correlated to *TP63* tissue-specific junction usage, we analyzed RNA-seq data from GTEx Analysis 8.0. The GTEx data are composed of 17,382 samples from 54 different tissues obtained from 980 donors. For each sample (*s*), the total number of reads (*Ts*) is calculated. The median read depth (*Mrd*) is calculated form each sample and a read depth normalization factor (*Ns*) for each sample is obtained as follows:

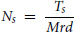


Normalized gene expression *Egs* for gene *g* and sample *s* is computed as:

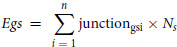


Tissues and samples expressing *TP63* are selected and used for correlation analysis. RNA-Binding Protein Database (RBPDB; ref. 30) provided a list of RBPs for which we calculated the RNA expression in the *TP63*-expressing samples. For each of the 21 *TP63* junctions, the junctionusage is computed as:

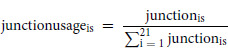


The Pearson correlation coefficient and associated *P* value are calculated between each RBP and the junctionusage of *TP63*. Top correlated RBPs are then selected for further analysis.

qRT-PCR and Western blots were statistically assessed with standard tests (ANOVA, *post hoc* Dunnet test, see Supplementary Table S2) using in-house R scripts. In all figures, error bars are SDs.

### Fluorescent RNA

Transcription templates were synthesized by PCR (GOTAQ G2, Promega) using a hybrid T7promoter/sequence-specific forward primer and a sequence-specific reverse primer. The PCR templates were either the WT minigene or the mutated ones. PCR products were checked on native agarose gel and purified on columns (DC5, Zymoresearch). Fluorescent RNAs were transcribed (37°C, 3 hours) in [40 mmol/L TRIS-Cl (pH7.9), 10 mmol/L NaCl, 6 mmol/L MgCl_2_, 2 mmol/L spermidine, 5 mmol/L DTT, rNTPs 0.5 mmol/L each, Cy3-UTP 0.1 mmol/L (Jena Bioscience), RNAsin (1 U/μL, Promega), T7 RNA polymerase (1.25 U/μL), DNA template (12.5 ng/μL)]. RNAs were purified on G50 sephadex (GE Healthcare) and controlled by denaturing electrophoresis and fluorescent detection (Typhoon FLA 9500). Cy3 fluorescence and RNA absorbance were quantified on a De Novix DS-11 spectro/fluorimeter.

### Protein Production and Electrophoretic Mobility Shift Assay

The DNA encoding hsPTBP1 (NM_002819.5) was obtained from source biosciences (IRAUp969B052D). The PTBP1 ORF was PCR amplified and subcloned by Gibson cloning into pET21A+ (Novagen) digested by *XhoI* and *NheI*. The 6xHis-tagged hsPTBP1 was expressed in *E.Coli* (BL21) after induction by IPTG (1 mmol/L), and purified on nickel column using standard procedures. PTBP1 was eluted with 250 mmol/L Imidazole and concentrated on vivaspin (30 kDa cutoff). Purified protein was resuspended in sodium cacodylate pH 7.0 20 mmol/L, NaCl 100 mmol/L, EDTA 0.5 mmol/L, DTT 1 mmol/L.

The electrophoretic mobility shift assay (EMSA) experiments were performed by mixing dilutions of hsPTBP1 in RNA binding buffer [RBB: Sodium Cacodylate pH 7.0 10 mmol/L, BSA 0.1 μg/μL, yeast tRNA 0.1 μg/μL, NaCl 100 mmol/L, MgCl_2_ 1 mmol/L, DTT 1 mmol/L, Rnasin (Promega) 0.4U/μL, Heparin (Sigma) 1U/μL] with an equal volume of labeled RNA in RBB. RNA protein complexes were analyzed on native polyacrylamide gels (31). Bound and free RNA were quantified on a Typhoon FLA 9500. To estimate the Kd, a nonlinear model of the form (bound/total RNA) − 1/(1 + Kd/[PTBP1]) was fitted to the data for each RNA using R script (32).

### PTBP1 Complex Immunoprecipitation

A total of 10 million HaCaT or SCC9 cells were lysed in (50 mmol/L Tris-HCl, 0.1 mol/L NaCl, 1% NP40, 0.1% SDS, 0.5% deoxycholate, protease inhibitor cocktail P8340 0.1%, RNAsin 400U, TurboDNAse 10U, DTT 1 mmol/L) and incubated 10 minutes at 37°C. Prior to immunoprecipitation, lysates were partially digested with RNAseT1 (Ambion; 0.25U per mg of lysate 5 minutes, 37°C). Lysates were precleared with 50 μL of dynabeads Protein-G magnetic for 1 hour at 4°C. The cleared supernatants were incubated with 50 μL of beads precoated with 40 μg of anti-PTBP1 antibodies (clone BB7) or mouse IgG Isotype Control (Invitrogen). Immunoprecipitations were conducted overnight at 4°C with constant shaking, then washed successively with IP300 (50 mmol/L HEPES-K pH 7.5, 300 mmol/L KCl, 0.05% NP-40, 0.5 mmol/L DTT), IP500 (50 mmol/L HEPES-K pH 7.5, 500 mmol/L KCl, 0.05% NP-40, 0.5 mmol/L DTT), IP750 (50 mmol/L HEPES-K pH 7.5, 750 mmol/L KCl, 0.05% NP-40, 0.5 mmol/L DTT) and finally once with Washing Buffer (20 mmol/L Tris-HCl pH 7.4; 10 mmol/L MgCl_2_; 0.2% Tween20).

One-tenth of the immunoprecipited samples were used for analysis of immunoprecipitated proteins by SDS-PAGE and Western blotting. Co-immunoprecipitated RNAs were isolated by proteinase K treatment (30 minutes, 37°C in 150 μL of PK buffer (50 mmol/L Tris pH 7.4, 1 mol/L NaCl, 0.5% NP40, 0.5 mmol/L EDTA, 0.1% SDS, 2 mol/L urea, 1% deoxycholate) containing 50 μg tRNA and 12 U proteinase K (Thermo Fisher Scientific) followed by phenol–chloroform extraction and ethanol precipitation. RNAs were analyzed by RT-PCR or qRT-PCR.

### Data Availability

All scripts are available from https://gitlab.com/YannAudic/tp63_tcga_gtex. Plasmids and transgenic cell lines are available upon request.

## Results

### Higher TP63 γ Exon Inclusion is Associated with Poor Outcome in Patients with HNSCC

While many HNSCC tumors overexpress *TP63* (Fig. 1B; ref. 33), the expression and impact of its C-terminal isoforms on patient survival is unclear. We used TCGASpliceSeq data to evaluate inclusion of exons composing the splice variants of *TP63* in patients with HNSCC. The *ΔNTP63* isoforms are characterized by transcription initiation at exon 4 (Fig. 1A; Supplementary Fig. S1A) and account for more than 99% of total *TP63* (Fig. 1C) in both normal and tumor samples. The *TATP63* isoforms initiated on exon 1 account for less than 1% of total *TP63*. Among the two terminal exons 12 and 16, the inclusion of exon 16 accounts for about 98% of the *TP63* RNA (isoforms α, β, or δ, see Supplementary Fig. S1B for isoform structure). Conversely, usage of exon 12, found exclusively in *TP63γ,* is weak (around 2% in average). A small but significant increase in the usage of exon 12 is observed in tumor samples compared with normal tissues (*P* = 0.010, Wilcoxon test) with a parallel decrease in exon 16 (Fig. 1C). The exclusion of exon 15 measures *TP63β*, while the exclusion of the pair of exons 14 and 15 measures *TP63*δ. No difference could be observed between tumors and normal sample for these splicing events. We sought to determine whether differential exon usage in tumors could be associated with difference in survival probability for patients (Fig. 1D). The exon 12 or exon 14+15 inclusion measurements in tumor samples could discriminate patient survival rates. Higher inclusion (PSI ≥ 4.07%) of the exon 12 was associated with a lower survival of patients (median half-life = 862 days compared with 2,319 days, *P* = 0.00363). Lower inclusion of exon 14+15 was also associated with a more moderate decreased survival (PSI < 98.5%, median half-life = 1,466 days compared with 2,319 days, *P* = 0.0177). When the analysis was restricted to human papillomavirus–negative patients, similar results were obtained (Supplementary Fig. S2). A higher inclusion of *TP63*γ exon appears to be detrimental for patient survival.

### Higher TP63 γ Exon Inclusion is Associated with Loss of Epithelial Markers in Primary Tumors of Patient with HNSCC

**FIGURE 2:**
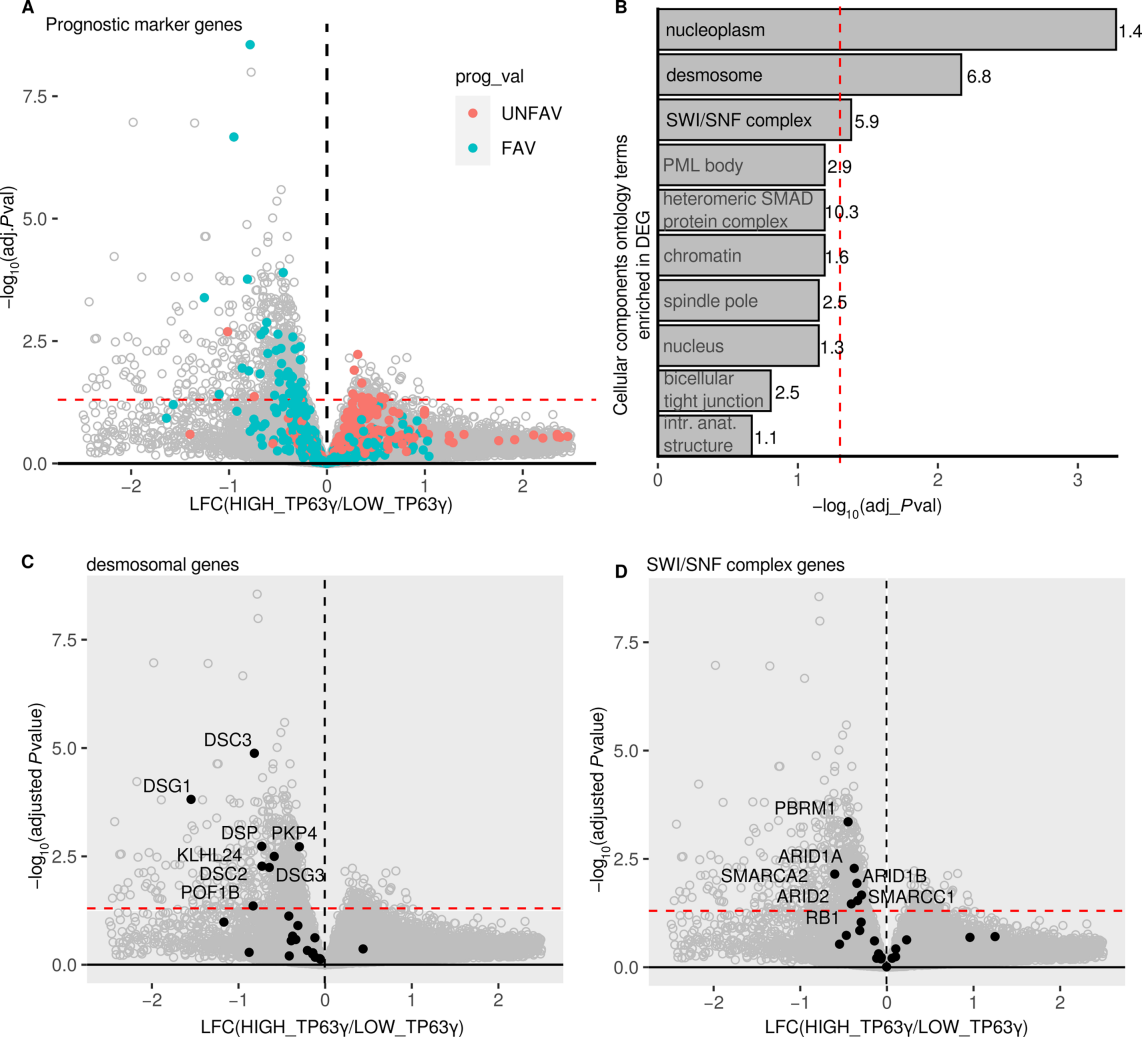
DEG and GO enrichment analysis of patients’ tumors with high TP63γ expression. **A,** Volcano plot of gene expression in the groups of patients with a high or a low percentage of *TP63γ* isoform. The red dashed lines represent the threshold at Benjamini–Hoschberg adjusted *P* value <0.05. The favorable and unfavorable prognosis marker genes are highlighted blue and red, respectively. **B,** GO term enrichment analysis performed on DEGs (*P*_adjusted_ < 0.05) shown in **A**, the enrichment (observed/expected) is shown on top of the bar. **C,** Same as **A** with desmosomal gene highlighted in black. **D,** Same as **A** with SWI/SNF complex gene highlighted in black.

On the basis of the results shown in Fig. 1A, we split the patients with HNSCC into two groups: those with a low percentage of *TP63*γ and a better prognosis, and those with a higher percentage of *TP63*γ and a worst prognosis. We identified the differentially expressed genes (DEG) between these two groups of patients and we highlighted the HNSCC prognostic marker genes. We observed that all prognostic marker genes overexpressed in the high *TP63γ* tumors are exclusively unfavorable (red dots, Fig. 2A). Conversely, prognostic genes overexpressed in the low *TP63γ* tumors are mainly favorable (blue dots, Fig. 2A). This is coherent with the decreased survival probability of high *TP63γ* patients. Ontology enrichment analysis of the DEG highlighted nucleoplasm genes, desmosomal genes, and SWI/SNF complex genes as statistically enriched (Fig. 2B; Supplementary Tables S3 and S4). The most significantly enriched term “nucleoplasm” relates to nuclear components that are affecting chromatin epigenetic modification, transcriptional regulation or replication. Most notably, eight of 24 genes encoding proteins of the desmosomal cell-to-cell adhesion complexes are less expressed in primary tumors of patients with high level of *TP63γ* (Fig. 2C). This is in line with the potential loss of epithelial characteristics that has been attributed to TP63γ (17, 34). Seven of 24 genes encoding proteins of the SWI/SNF complex involved in the extensive remodeling of the chromatin are downregulated in the same primary tumors (Fig. 2D), in accordance with a loss of function of the SWI/SNF complex that is often associated with carcinogenesis (35). This obviously raises the question of the regulators of *TP63* splicing in different patients.

### Candidate RBPs Controlling TP63 γ Exon Inclusion

**FIGURE 3:**
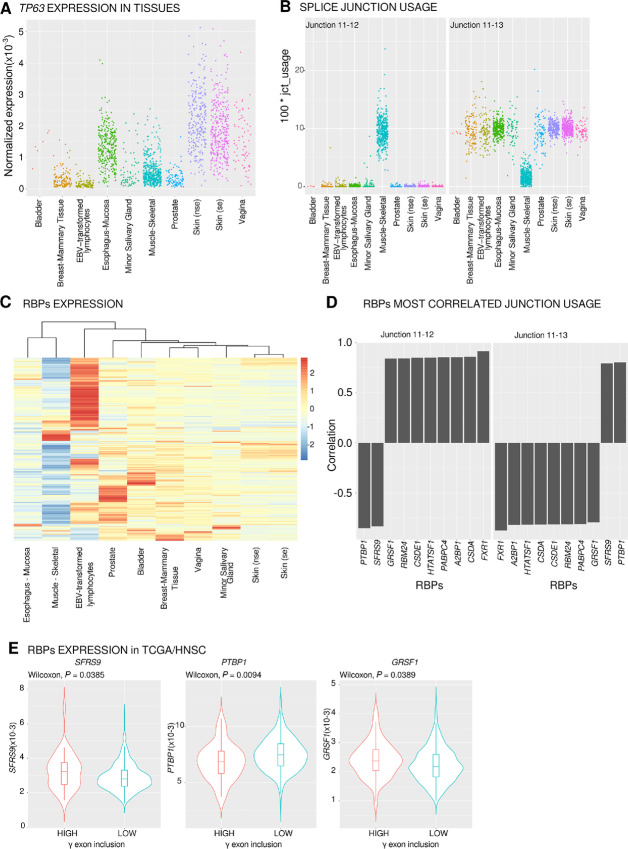
Identification of *PTBP1* as an RBP correlated to *TP63γ* terminal exon splicing. **A,***TP63* gene expression in samples from *TP63*-expressing tissues (normalized reads count). se, nse, (non) sun exposed. **B,** Quantification of the usage of junctions 11–12 and 11–13 pertinent to γ-terminal exon inclusion. **C,** RBP expression heatmap and hierarchical clustering of tissues based on RBP expression from GTEX data. **D,** Top 10 RBPs most correlated to the junctions involved in γ exon inclusion (junction 11–12) or γ exon exclusion (junction 11–13). **E,** Expression of *SFRS9*, *PTBP1,* and *GRSF1* in two classes of patients with high or low γ exon 12 inclusion in tumors (threshold = 4.07%).

Because tumor samples may be highly heterogeneous due to tumor type, location, and/or intermixed tissues, we sought to identify potential regulators of *TP63* splicing using data from normal tissues. We searched for RBPs whose expression correlates to *TP63* junction usages. RNA-seq junction quantification was obtained from the GTEX consortium (36). They represent quantifications from 54 different tissues or conditions. The correspondence between GTEX and TCGA nomenclatures for *TP63* is shown in Supplementary Fig. S1A and a table ascribing the different transcripts relative to the ENSEMBL annotation is in Supplementary Fig. S1C. We calculated the expression of *TP63* in each sample and for all tissues (see Materials and Methods). Among the 54 tissues, 10 (10/54, 18.5%) were selected as expressing *TP63* (Fig. 3A). These tissues are mainly of epithelial origin, as expected for *TP63*, but Epstein-Barr Virus (EBV)- transformed lymphocytes and skeletal muscles also showed a notable *TP63* expression.

For each tissue and sample, we quantified the usage of the 21 individual *TP63* junctions (Supplementary Fig. S3A). The junction usage is strikingly different between tissues (Supplementary Fig. S3B). We can distinguish three groups of tissues: muscle, epithelia, and EBV-transformed lymphocytes. Eleven junctions are used at similar levels across tissues. Among those, four are very weakly used (J4-6, J5-5′, J5’-6, and J13-16) and seven are constitutive splicing events included at high levels in all tissues. Ten junctions are differentially used across tissues. Junctions (J1-2, J2-3, J3-5, J4-5) are representative of differential promoter usage between epithelial tissue (ΔN isoforms favored) and muscle and EBV-transformed lymphocytes (TA isoforms favored). Muscle specific differences (J11-12 and J11-13, red boxes) are visible. These differences are not observed in EBV-transformed lymphocytes. The competition between these two mutually exclusive splicing reactions (J11-12, J11-13) sharing an identical 5′SS allows for the production of the γ or (α, β, δ) terminal isoforms. The quantification of these two reactions (Fig. 3B) clearly shows that they are oppositely regulated: epithelial tissues favor the exclusion of the γ exon, while oppositely about 85% of the *TP63* transcripts include the γ terminal exon in skeletal muscle (Supplementary Fig. S4).

We hypothesized that correlations between RBP expression and junction usage could allow us to pinpoint RBPs acting as splicing enhancers or splicing silencers affecting the tissue-specific inclusion of the γ exon. Using the RBPs represented in the RBPDB, we first determined their expression in our selected *TP63*-expressing tissues. We were able to analyze the expression levels of 386 different RBPs in these tissues (Fig. 3C). On the basis of individual samples, we computed the correlations between RBP expression and junction usage relevant to the γ exon (J11-12 and J11-13). Among the 10 most correlated RBPs (Fig. 3D), eight were positively correlated and two were negatively correlated to γ exon inclusion (J11-12). As expected, the correlation was inverted when considering the alternative junction (J11-13). On the basis of the above analysis of the expression levels of 386 RBPs, the 10 most correlated RBPs are strongly expressed in muscle (*FXR1, GRSF1, RBM24, CSDE1/UNR, HTATSF1, PABPC4, A2BP1/RBFOX1, CSDA/YBX3*) or in epithelial tissues (*PTBP1, SRSF9*).

To determine whether expression of the selected RBPs is coherent with higher γ exon inclusion in patients with HNSCC with poor survival, we compared the differential expression of the RBPs in our previously established tumor groups selected for having high or low levels of γ exon which are respectively associated with lower or higher patient survival (Fig. 1C). Of the 10 RBPs tested, only three (*SFRS9, PTBP1, GRSF1*) are differentially expressed at the RNA level between tumors from patients with high or low γ exon inclusion (Fig. 3E, Wilcoxon, *P* < 0.05). *SFRS9* expression is higher in samples with high γ exon inclusion (Wilcoxon, *P* = 0.0385), which is not in accordance with the GTEX data where *SRSF9* expression is negatively correlated to γ exon inclusion (Fig. 3D). *PTBP1* and *GRSF1* are respectively low and high in patients with high *TP63γ* (Wilcoxon, *P* = 0.0094 and *P* = 0.0389; Fig. 3E). The same RBPs are negatively and positively correlated, respectively, with γ exon usage in GTEX data (Fig. 3D). Therefore, PTBP1 and GRSF1 appear to be plausible candidates for γ exon regulation. However, because GRSF1 is mainly a mitochondrial RBP and is weakly expressed in epithelial cells (37) whereas PTBP1 is a well-defined alternative splicing regulator expressed in epithelial tissues, we chose to test whether experimental depletion of PTBP1 could enhance *TP63* γ exon inclusion in a panel of HNSCC cell lines of different common tumor locations (FaDu, SCC9, SCC25, Detroit562, and A253).

### PTBP1 Represses Endogenous *TP63 γ* Exon Inclusion in Squamous Carcinoma Cell Lines

**FIGURE 4:**
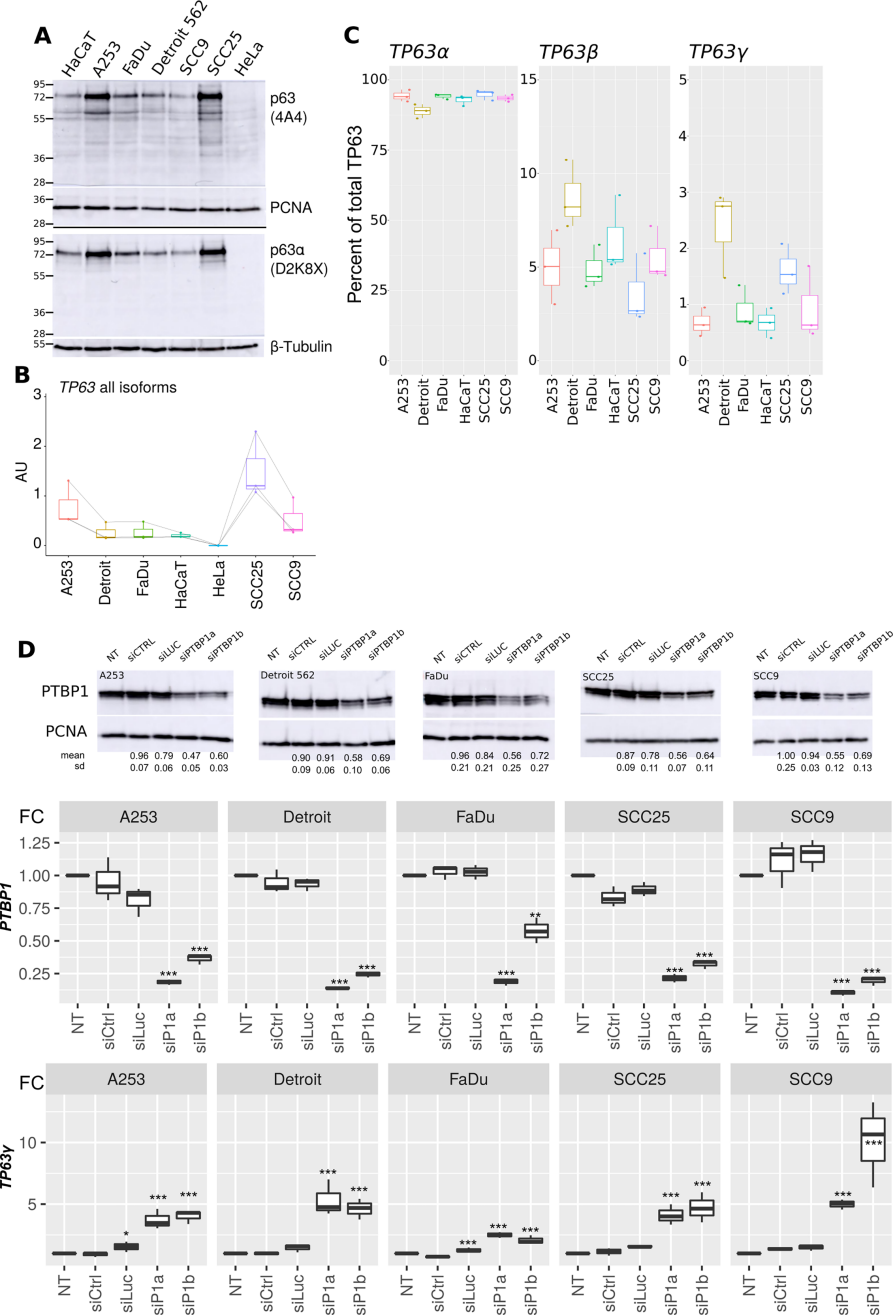
PTBP1 controls *TP63* splicing in HNSCC cell lines. **A,** The abundance of p63 protein was evaluated in HaCaT, HeLa, and HNSCC cells by Western blot analysis and immunodetection using a pan-p63 antibody (4A4) or a p63α-specific antibody (D2K8X; representative results of three independent replicates). Even loading was controlled using proliferating cell nuclear antigen (PCNA) and β-tubulin antibodies as indicated. **B,** Total *TP63* RNA was measured by qRT-PCR with one pair of primers detecting all isoforms. Quantification was normalized to a calibration curve obtained from *TP63* plasmid DNA dilutions (*n* = 3). **C,** Quantification of *TP63*α, *β,* or *γ* proportions using isoform-specific primer pairs (*n* = 3). The amount of each isoform was normalized by the total abundance of *TP63* mRNA as in **B**. **D,** Top, Evaluation of PTBP1 depletion by Western blot analysis, PCNA serves as a loading control. Remaining PTBP1 levels in the three independent experiments are presented below each panel. Bottom, Quantification of *TP63*γ isoform and *PTBP1* RNA in control and PTBP1-depleted HNSCC cell lines (*n* = 3). *, *P* < 0.05; **, *P* < 0.01; ***, *P* < 0.001; Dunnett test.

Using two different antibodies, we analyzed total p63 and p63α expression by Western blot analysis (Fig. 4A) in a panel of HNSCC cell lines. Both antibodies detected a major isoform around 70 kDa, coherent with the major ΔNp63α isoform (MW 66 kDa). In addition to the 70 kDa protein, the pan-p63 antibody (4A4) detected lower molecular weight bands undetected using the α-specific antibody (D2K8X). This suggests that isoforms other than ΔNp63α are present at lower levels in the tested cells. The identity of these isoforms cannot be inferred from these experiments. In parallel, we used qRT-PCR to evaluate the abundance at the RNA level of total *TP63*. We estimated that global *TP63* expression levels are highest in SCC25, then A253 and SCC9, then FaDu, Detroit 562, and premalignant keratinocytes HaCaT. As expected from the human protein atlas and the Western blot analysis (Fig. 4A), *TP63* is only faintly detectable in HeLa cells used as a negative control (Fig. 4B). We used isoform-specific qRT-PCR to quantify mRNAs encoding the three predominant (α, β, γ) C-terminal isoforms. Figure 4C shows the proportions of each isoform, relative to total *TP63*. In all cell types tested, the α isoform was the most abundant ranging from 87% to 95% of total *TP63* mRNA. Detroit 562 cells have the lowest α proportion. Accordingly, β and γ isoforms are in higher proportions in Detroit 562 cells with 8% and 2.7% of each, respectively. The abundances of the β and γ isoforms are variable among the cell lines with the Detroit 562 and the SCC25 cells having the highest relative proportion of *TP63γ* (2.8% and 1.5%, respectively) while other cell lines have all less than 1% of *TP63γ* mRNA (Fig. 4C).

We reduced PTBP1 levels in the five HNSCC cell lines using two different siRNAs. The siPTBP1a was more efficient to deplete PTBP1 as can be seen from the Western blot analysis (Fig. 4D, top), the qRT-PCR (middle), and from the increased expression of *PTBP2* which is known to be under PTBP1 repression (ref. 38; top, Supplementary Fig. S5). Yet, presumably because of an existing posttranscriptional regulatory loop controlling PTBP1 protein levels (39), achieving full depletion of PTBP1 was not possible. In all five cell lines, depletion of PTBP1 led to a significant increase in *TP63γ* (Fig. 4D) and a decrease in *TP63β* abundance (bottom, Supplementary Fig. S5) without affecting total *TP63* or *TP63α* levels (middle, Supplementary Fig. S5). The *TP63γ* levels are therefore inversely related to PTBP1 levels. This demonstrates that PTBP1 represses the accumulation of the *TP63γ* isoform in all five HNSCC cell lines tested.

### PTBP1 Binds to *TP63* Pre-mRNAs Sequences *In Vivo* on the γ Exon 3′SS and CPAs

**FIGURE 5:**
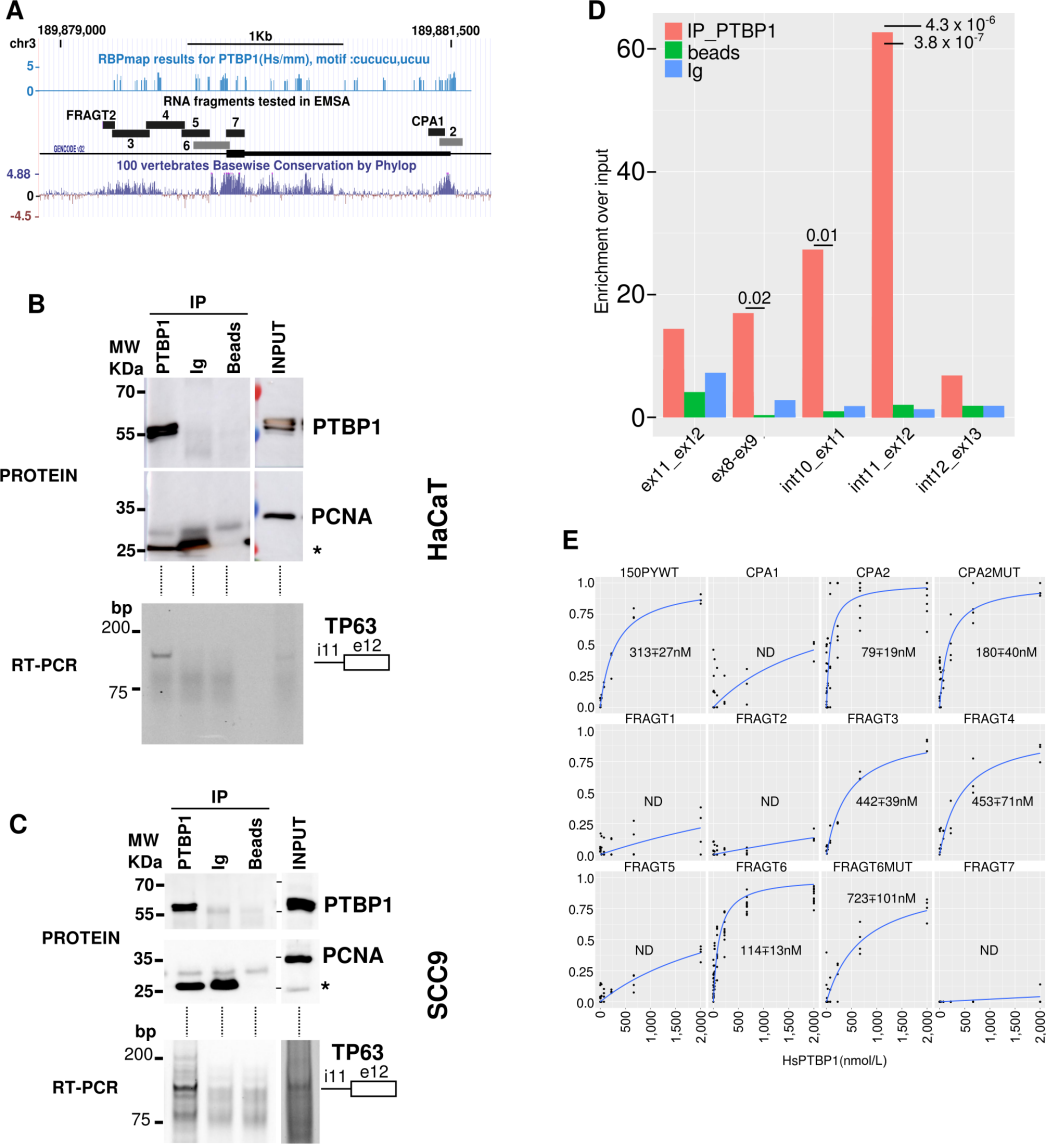
PTBP1 binds specifically to *TP63* premRNA sequences. **A,***TP63* exon 12 genomic locus and positions of the RNAs tested in RNA/protein shift assays. Top lane, RBPmap predicted PTBP1 binding sites. Middle, Positions and names of the RNA fragments tested along the *TP63* exon 12 genomic sequence. Bottom lane, nucleotidic conservation among vertebrates. **B,** Immunopurification of PTBP1/RNA complexes or control experiments (Ig and beads only) from HaCaT cell extracts. Immunopurification was controlled by PTBP1 and PCNA Western blot analysis as indicated in input (10%) or immunoprecipited (IP) samples (top). Protein MW are indicated on the side of the membranes. The asterisk denotes the light chain of the IgG. Bottom, RT-PCR detection of *TP63* pre-mRNA with primers located in intron 11 and exon 12. **C,** Same as **B** from SCC9 cell extracts. **D,** qRT-PCR detection in HaCaT cells of different mRNA or pre-mRNA portions of *TP63* from IP or control samples relative to input signal. Statistical assessment between PTBP1 and control IP was measured by a Student *t* test (*n* = 3). **E,** PTBP1/RNA-binding curves [bound/(bound + free)] obtained for the indicated RNAs (*n* ≥ 4). RNA mutated to remove PTBP1 binding sites are shown in gray in **A**. The calculated Kd and their SD is shown for each plot.

The control of *TP63* splicing by PTBP1 could be direct or indirect. Direct regulation of *TP63* splicing would require binding of PTBP1 to regulatory elements located in the pre-mRNA of interest. *In silico* prediction by RBPmap (40) revealed several putative PTBP1 binding sites within *TP63* pre-mRNA (Fig. 5A, top). Interestingly, these sites are enriched within regions that are evolutionary conserved in vertebrates (Fig. 5A, bottom). This prompted us to examine whether the potential regulation of *TP63* by PTBP1 is also evolutionarily conserved, which would highlight its fundamental importance. The exon/intron structure of the *TP63* locus is well conserved between *Xenopus laevis* and human with an internal terminal exon corresponding to the human γ exon present in both species (Supplementary Fig. S6A). In frog embryos (*Xenopus laevis*), the depletion of Ptbp1 leads to an increase of *tp63γ* mRNA as shown by RNA-seq (Supplementary Fig. 6B) and qRT-PCR (Supplementary Fig. S6C), and this effect is reversed by reintroducing Ptbp1 (Supplementary Fig. S6C). Hence, the PTBP1-dependent regulation of *TP63γ* production is a conserved phenomenon that was already present in the common ancestor of Mammals and Amphibian roughly 360 million years ago (41).

**FIGURE 6:**
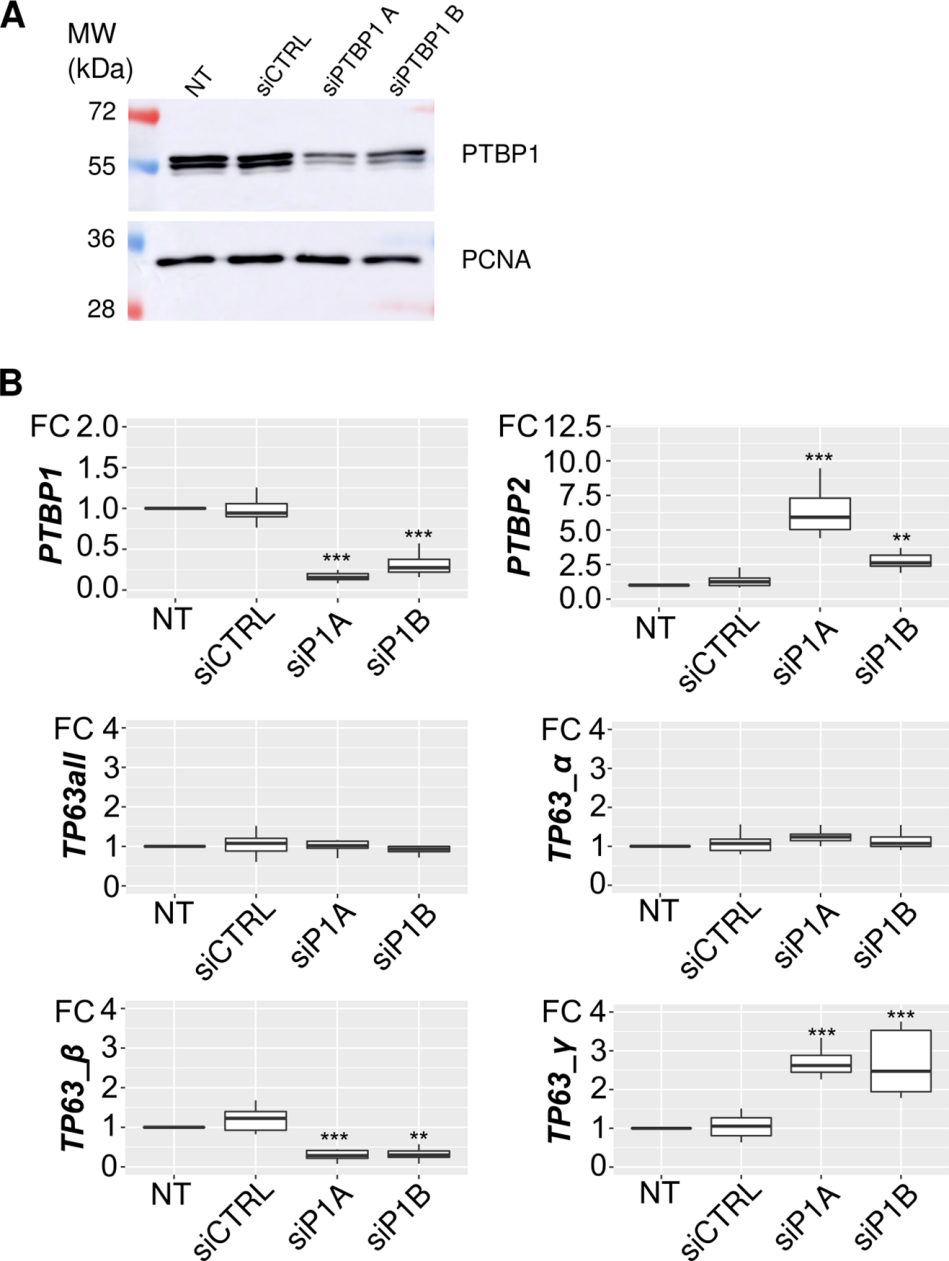
PTBP1 represses endogenous *TP63 γ* exon inclusion in HaCaT cells. **A,** Detection of PTBP1 in HaCaT cells after PTBP1 depletion mediated by two different siRNA. We show here a representative experiment of a triplicate. **B,** qRT-PCR quantification of endogenous *PTBP1*, *PTBP2*, *TP63all* and *TP63α*, *β,* and *γ* isoforms levels after siRNA-mediated PTBP1 depletion. *n* = 3; *, *P* < 0.05; **, *P* < 0.01; ***, *P* < 0.001; Dunnett test (*n* = 3, Dunnet test).

To confirm *in vivo* that PTBP1 binds to *TP63* pre-mRNAs, we immunoprecipitated specifically PTBP1/RNA complexes from keratinocytes (HaCaT) or squamous cell carcinomas cell lines (SCC9) cell extracts and analyzed the pre-mRNA content of the complexes by RT-PCR (Fig. 5B and C). The presence of the *TP63* pre-mRNA was specifically detected in input and PTBP1 immunoprecipitates obtained from both cell lines, but not using control Ig or beads only. To determine whether PTBP1 was preferentially associated to *TP63* pre-mRNA or mRNA, we ran qRT-PCR with primers specific to *TP63* mRNAs (ex8-ex9, ex11-12) or pre-mRNA (int10-ex11, int11-ex12, int12-ex13) in HaCaT cells. As shown in Fig. 5D, the most significant and robust enrichment was observed with the primer pair specific to the pre-mRNA region closest to the γ exon (int11-ex12). Control RT-qPCR performed in absence of reverse transcriptase did not show PCR amplification (Supplementary Fig. S7A). Therefore, on *TP63* pre-mRNA, PTBP1 is preferentially bound to the region that is upregulated following PTBP1 depletion.

**FIGURE 7:**
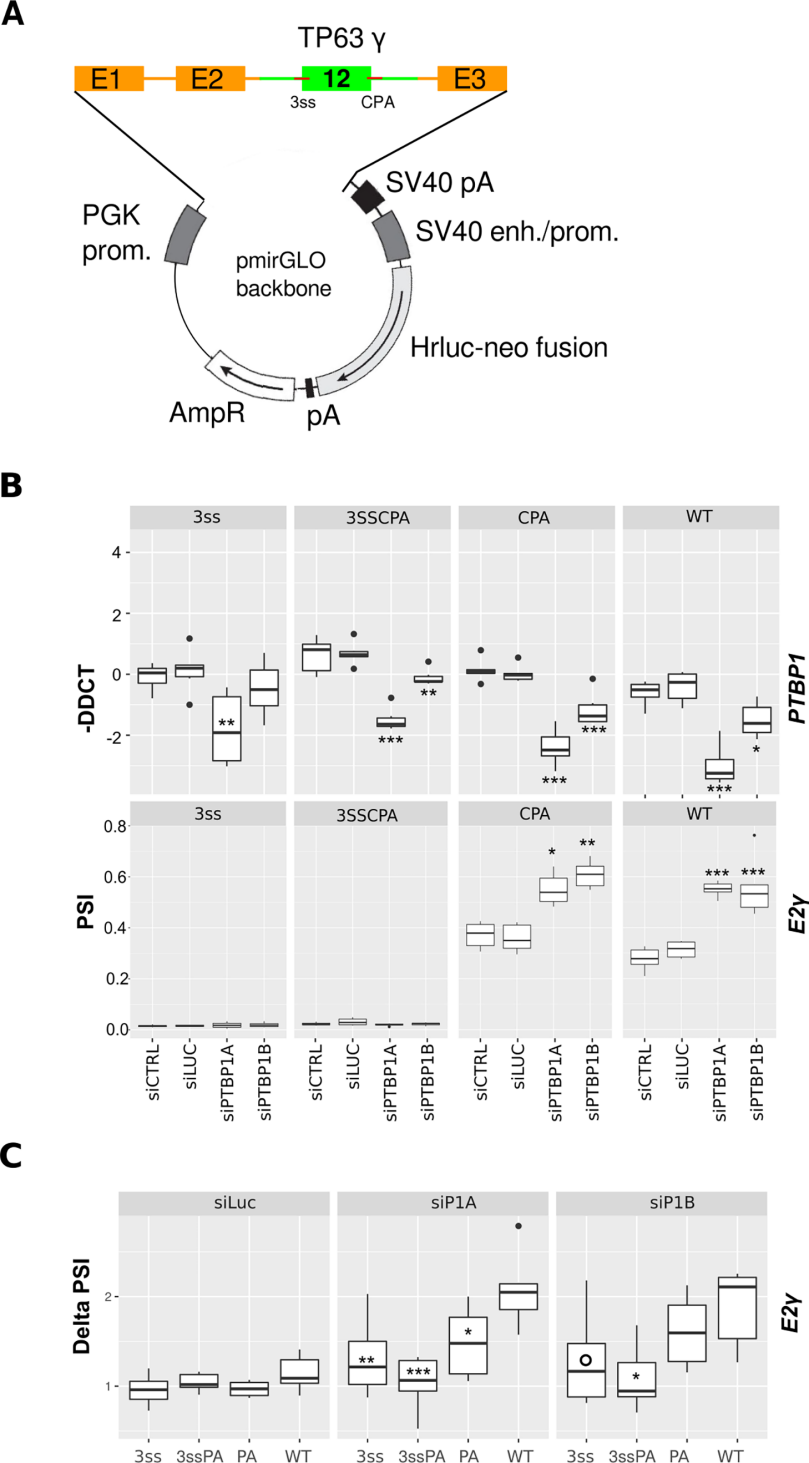
A *TP63γ* minigene recapitulates the PTBP1-dependent regulation of exon γ inclusion. **A,** Structure of the *TP63* minigene stably transfected in HaCaT cells. β-globin gene sequences are shown in orange, *TP63* gene sequences are shown in green. **B,** qRT-PCR quantification of *PTBP1*, and the RNA isoforms produced from the *TP63* minigene after siRNA-mediated PTBP1 depletion. **C,** Delta PSI are computed as the fold difference between test and siCTRL as the reference condition. In all panels, *n* = 6; *, *P* < 0.05; **, *P* < 0.01; ***, *P* < 0.001; Dunnett test.

To determine the affinity of PTBP1 for TP63 pre-mRNA sequences, we performed EMSA using recombinant PTBP1 (Supplementary Fig. S7) and several fluorescently labeled RNA molecules that cover *TP63* pre-mRNA in the vicinity of exon *γ* (see Fig. 5A for the location of the RNA fragments). Representative EMSA experiments are shown in Supplementary Fig. S7B, and the binding curves for at least four replicate experiments per RNA are presented in Fig. 5E. This allowed us to determine the Kd of PTBP1 for these RNAs (32). As presented in Fig. 5E, RNA fragment 1 (FRAGT1) is composed solely of β-globin RNA sequences, FRAGT2 is composed in part of β-globin RNA sequences and in part of the *TP63* intronic region, FRAGT5 is located entirely in the intronic region upstream of the γ exon, FRAGT7 is fully located within the γ exon and CPA1 is located close to the CPA, they all remain unbound to PTBP1. FRAGT3, FRAGT4, FRAGT6, and CPA2 associate strongly with human PTBP1 protein with binding affinities better than (CPA2, FRAGT6, − 60 nmol/L) or comparable with (FRAGT3, FRAGT4, − 150 nmol/L) the positive PTBP1 binding control RNA (150PYWT, − 104 nmol/L; ref. 42). Mutating the UCU or UCUU motifs to CCC or CCCC in FRAGT6 (FRAGT6MUT) drastically decreased PTBP1’s affinity for it (6-fold increase of the Kd, compare FRAGT6 and FRAGT6MUT). In the CPA2 RNA, we altered the PTBP1 motif to a UGUU motif rather than CCCC to keep the UG richness required for the activity of the downstream element of the polyadenylation signals (CPA2MUT). This mutation provoked a 2-fold decrease in the affinity of PTBP1 for this sequence (Fig. 5E).

Altogether, this demonstrates the direct and sequence-specific association of PTBP1 with regulatory regions surrounding the γ exon 3′SS and CPA on the *TP63* pre-mRNA *in vivo* in both a noncancerous keratinocyte cell lines (HaCaT) and a HNSCC cell line (SCC9).

### PTBP1 Represses *TP63 γ* Exon Inclusion in a Minigene in Epithelial Cells

If PTBP1 can indeed control *TP63γ* exon inclusion by directly binding to cis-elements within the pre-mRNA, then the splicing of a minigene composed of the *TP63* genomic sequence should be under the control of PTBP1. To test this, we stably transfected HaCaT cells with minigenes containing *TP63* genomic sequences within an efficient *β-globin* splicing context. We first tested whether the PTBP1-dependent regulation we discovered in HNSCC cell lines was acting in HaCaT cells on endogenous *TP63*. We independently depleted PTBP1 in HaCaT cells with two different siRNAs as above. PTBP1 depletion strongly increases the abundance of *TP63 γ* exon and decreases levels of *TP63β* in HaCaT cells without affecting global *TP63 (TP63_all)* or *TP63α* mRNA levels (Fig. 6A and B) as we previously showed in HNSCC cell lines (Fig. 4D). This demonstrates that, like in HNSCC cell lines, the accumulation of the endogenous *TP63γ* isoform is repressed by PTBP1 in HaCaT keratinocytes.

We generated four HaCaT cell lines stably expressing *TP63* minigenes in WT and mutated versions. The mutated variants of the minigenes harbor the mutations depicted in the gel shift experiment (Fig. 5E) on the 3′SS, the CPA or both (3ssCPA). The structure of the minigene is shown in Fig. 7A. When expressed in the cells, measuring splicing of the junction of exon E2 to exon γ (E2γ) accounts for the production of the γ isoform. We decreased PTBP1 levels (RNA levels are shown in Fig. 7B and protein levels in Supplementary Fig. S8A) in these minigene-expressing HaCaT cell lines. We quantified endogenous *TP63* isoforms (Supplementary Fig. S8B) and *TP63* minigene splicing by qRT-PCR (Fig. 7B). It is worth noting that the PTBP1-dependent regulation of the endogenous *TP63* RNA is identical in all four HaCaT cell lines (Supplementary Fig. S8B). The WT *TP63* minigene produces about 30% of E2γ RNA as quantified by qRT-PCR (Fig. 7B, PSI). Mutations of the PTBP1 binding sites close to the 3′SS completely abolish the splicing between E2 and the γ terminal exon (Fig. 7B, 3SS, 3SSCPA) compared with the WT or CPA minigene. This probably indicates that the replacement of all the UCU/UCUU motifs by CCC/CCCC sequences drastically affect the recognition of the 3′SS signal of the terminal γ exon. In the siCTRL or siLUC conditions, the minigene harboring mutations of PTBP1 binding sites on the CPA alone produced 40% of E2γ RNA, to compare to the 30% inclusion from the WT minigene.

**FIGURE 8:**
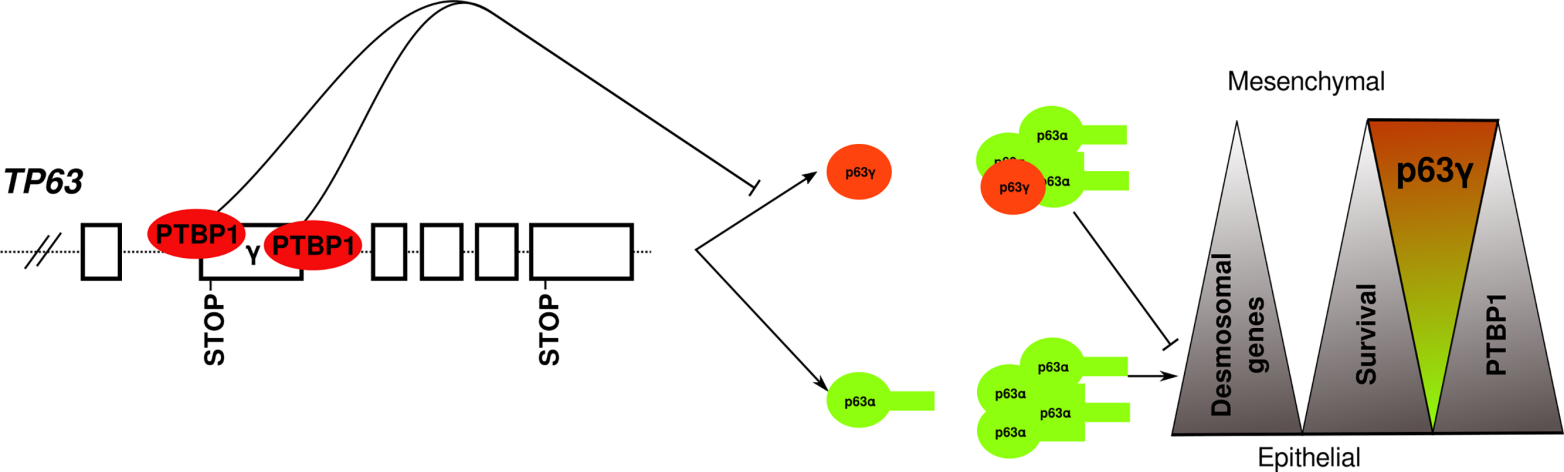
PTBP1-dependent regulation of TP63γ in HNSCC*.* Graphical abstract of the main conclusion and hypothesis. Left side, PTBP1 by binding on 3′SS and CPA of the alternative terminal exon *γ* repress its inclusion in the mature RNA. Right side, upon decreased PTBP1 expression, accumulation of *TP63γ* promotes formation of TP63 α/γ heterotetramers that repress desmosomal genes expression in primary tumors, alter epithelial characteristics, and promote an unfavorable prognostic.

Upon depletion of PTBP1, the 3′SS and 3SSCPA mutant remained unaffected, in agreement with the mutated 3′SS being inoperative. Both the WT and the CPA mutant are responsive to PTBP1 depletion. Following PTBP1 depletion, the splicing between exon E2 and the γ terminal exon increased about 2-fold in both the WT and the CPA mutant minigenes (Fig. 7B). To better assess the amplitude of the PTBP1-dependent regulation, we plotted a Delta PSI value calculated as the fold difference of exon γ inclusion between treatment conditions and the reference condition (siCTRL). We then tested whether there were any significant differences between the Delta PSI of the WT minigene and that of the mutated minigene. As can be observed on Fig. 7C, both siRNAs targeting PTBP1 promoted efficient inclusion of the γ exon on the WT whereas the control siLuc treatment barely changed inclusion of E2γ for the different minigenes. Interestingly, a decreased dynamic of exon γ inclusion on the CPA minigene could be observed in the most potent conditions of PTBP1 depletion (Fig. 7C, SiP1A). This indicates that the PTBP1-binding site close to the CPA contributes to PTBP1-mediated repression of exon γ inclusion.

These experiments demonstrate that the genomic regions surrounding the γ terminal exon are sufficient to allow for its PTBP1-dependent regulation. Mutational analysis demonstrates that the PTBP1 binding sites are intermingled with elements that define the 3′SS and that mutations over the CPA region that decrease the affinity of this region for PTBP1 can promote a better inclusion of the γ exon in control conditions. Mutations that partially reduce the affinity of PTBP1 for the CPA render the minigene less sensitive to PTBP1 depletion.

As summarized in Fig. 8, these data reveal that in HNSCC, PTBP1 controls *TP63γ* exon inclusion by binding to discrete and conserved regulatory elements located in this pre-mRNA region. In normal and cancerous epithelial cell lines where PTBP1 is abundantly expressed, it reduces the inclusion of the γ exon and therefore decreases the abundance of the TP63γ isoform. The increase of *TP63γ* isoform is correlated to a repression of some epithelial marker genes and to a decreased survival probability of patients with HNSCC.

## Discussion

Alternative splicing misregulation is emerging as a major theme in the alteration of gene expression observed during the cancerous process (43) and offers new routes of tumor classification or treatment (44). Here, we identify for the first time an RBP that controls the balance between different splice isoforms of *TP63*, a key gene involved in squamous cell carcinoma and especially HNSCC. We demonstrate using a variety of approaches that PTBP1 inhibits the synthesis of the *TP63γ* isoform. PTBP1 is generally known as an inhibitor of the splicing or polyadenylation reactions. The multiple RNA-recognition motifs of PTBP1 and its possible multimerisation on its targets offer multiple modes of interaction (45). PTBP1 can cause internal exon skipping by binding to the pre-mRNA on either side of the regulated exon, creating a loop that impairs spliceosome recognition (46) PTBP1 also regulates tandem CPA usage, which corresponds to the situations where several consecutive CPAs are present on the same terminal exon (47). Here, we reveal a third type of splicing event that is controlled by PTBP1, where a terminal exon (exon γ*)*, defined by a 3′SS and a CPA, is in competition with a downstream classical internal exon defined by a 5′SS and a 3′SS. We show that PTBP1-binding regions are present on either side of exon γ*.* Interactions between splicing factors involved in the recognition of the 3′SS and the CPA bound factors are central in the efficient processing of terminal exon (48). We therefore propose that by binding to both the 3′SS and the CPA, PTBP1 interferes with the ability of trans-acting factors to interact with their target sequences and therefore impedes their ability to define the γ exon.

The regulation of *TP63* splicing, which is mediated by PTBP1, has strong consequences in HNSCC. We show by analysis of TCGA data that *TP63γ* is associated with poor HNSCC patient outcome. This suggests that the different isoforms of p63 can have different functions in cancer cells. The genomic structure of the *TP63* gene is evolutionarily conserved. It is also at least partly the case for the regulation of *TP63* splicing, as we show here that PTBP1 similarly controls *TP63γ* production in humans and *Xenopus*. However, only 15/37 (40.5%) amino acids of the γ-specific peptides are conserved between these two species, while the overall identity of ΔNp63α proteins is 85%. This strong divergence makes it unlikely that the γ-specific peptide has any conserved functions by itself. Rather, the putative specific functions of the p63γ isoform probably rely on the lack of the 231 amino acids that are present in the C-terminal region of p63α. p63 forms homotetramers or heterotetramers composed of various p63 or p73 proteins (49). We can hypothesize that the p63γ isoform, because it is devoid of the TA, SAM and TID domains, affects the activity of the p63 tetramer in a dominant-negative manner (50).

Even in patients classified as expressing “high” levels of *TP63γ*, the proportion of that isoform relative to the other isoforms and especially *TP63α* is only a few percent. How then can this low amount act as dominant negative? First, the ability of an alternative isoform to affect the function of a protein complex generally increases with the number of monomers composing the complex (51). Second, the moderate amount of *TP63γ* RNA does not necessarily imply such a low p63γ protein level. Expression experiments showed that ΔNp63γ is the most stable p63 isoform in Hep3B cells (50). This may be due to ΔNp63γ being deprived of the Fbw7 E3 ligase PD and of some of the lysine residues (K494, K505) involved in ubiquitinylation and destabilization of p63α (12, 52). Being able to quantify the γ isoform at the protein level would therefore be of high interest. Unfortunately, no γ-specific antibody is currently available. State-of-the-art mass spectroscopy may allow for isoform-specific quantification of protein in the near future and solve the important question of the amount of p63γ in cancer cells (53). In patients, an interesting alternative could be the detection of potential isoform-specific autoantibodies, as recently shown for another *TP53* family member, *TP73*, in patients with colorectal carcinoma (54).

As a likely dominant negative of a transcription complex, p63γ very probably shapes the transcriptome of cancer cells. This prediction will have to be tested by transcriptomic approaches following manipulations of p63γ levels in cultured cells. However, correlation analyses in TCGA datasets already revealed putative targets of p63γ, such as genes encoding component of the chromatin remodeling complex SWI/SNF, or even direct p63 target genes as the ones encoding components of the desmosomes*.* Mutations in nine different SWI/SNF genes are collectively found in 25% of all cancer types (35). These mutations are frequently postulated to be loss-of-function mutations (55). We observe that, in patients’ tumors with a high proportion of *TP63γ* isoform, six SWI/SNF genes belonging to the ones found mutated in HNSCC are repressed. This suggests that downregulation of SWI/SNF genes can be part of the disease process in patients with a high proportion of *TP63γ* and a poor prognosis, with a potential for therapeutic opportunities (reviewed in ref. 56). The expression of most desmosomal genes is also reduced in primary tumors of patients with high *TP63γ*. Finding that p63γ potentially represses the expression of desmosomal genes is in agreement with nonfunctional p63 variants where mutations are located mainly in the C-terminal region of p63 and are responsible for Ankyloblepharon Ectodermal Defects Cleft lip/palate associated with desmosomal gene repression (57). Loss of desmosomes is an indication of the loss of epithelial characteristics of these primary tumors. This is consistent with the impact of p63γ on promotion of a more mesenchymal state, as demonstrated in a breast cancer cell line (34) and in a three-dimensional organotypic model of invasion (17). EGFR inhibitors such as cetuximab are used in patients with late-stage HNSCC. They stimulate the reexpression of desmosomal genes (58). However, only a subset of patient with HNSCC respond to EGFR inhibitors and no specific feature can predict which ones (59). Together, these and our observations make it tempting to speculate that the *TP63γ* isoform levels may allow for the definition of patients that could benefit from an early use of EGFR inhibitors.

While our work identified regulatory elements and factor controlling *TP63γ* production, much work remains needed to fully address the transcriptional functions and physiopathologic consequences of this conserved C-terminally truncated isoform of TP63.

## Supplementary Material

Supplementary Table 1 Reagents table

Supplementary Table 2 Results of the dunnet's tests presented in the figures

Supplementary Table 3 Differential gene expression

Supplementary Table 4 GO term enrichment analysis

Figure S1 TP63 gene structure and annotation

Supplementary Figure 2 TP63 isoforms splicing in patients segregated by HPV status

Supplementary Figure 3 Alternative junction usage for TP63 in GTEX data

Supplementary Figure 4 Terminal TP63 gamma exon in tissues

Supplementary Figure 5 PTBP1 controls TP63 splicing in HNSCC, additional RTqPCR

Supplementary Figure 6 Conservation of TP63 splicing regulation by PTBP1 in vertebrates

Supplementary Figure 7 RIP controls and EMSA

Supplementary Figure 8 endogenous TP63 regulation is unaffected in HaCaT cell lines expressing minigenes
