# Supplementary figures and images for: The Splicing Factor PTBP1 Represses TP63 γ Isoform Production in Squamous Cell Carcinoma
Source: Cancer Res Commun. 2022 Dec 20;2(12):1669–83. doi: 10.1158/2767-9764.CRC-22-0350 (PMC10035508; doi:10.1158/2767-9764.CRC-22-0350)

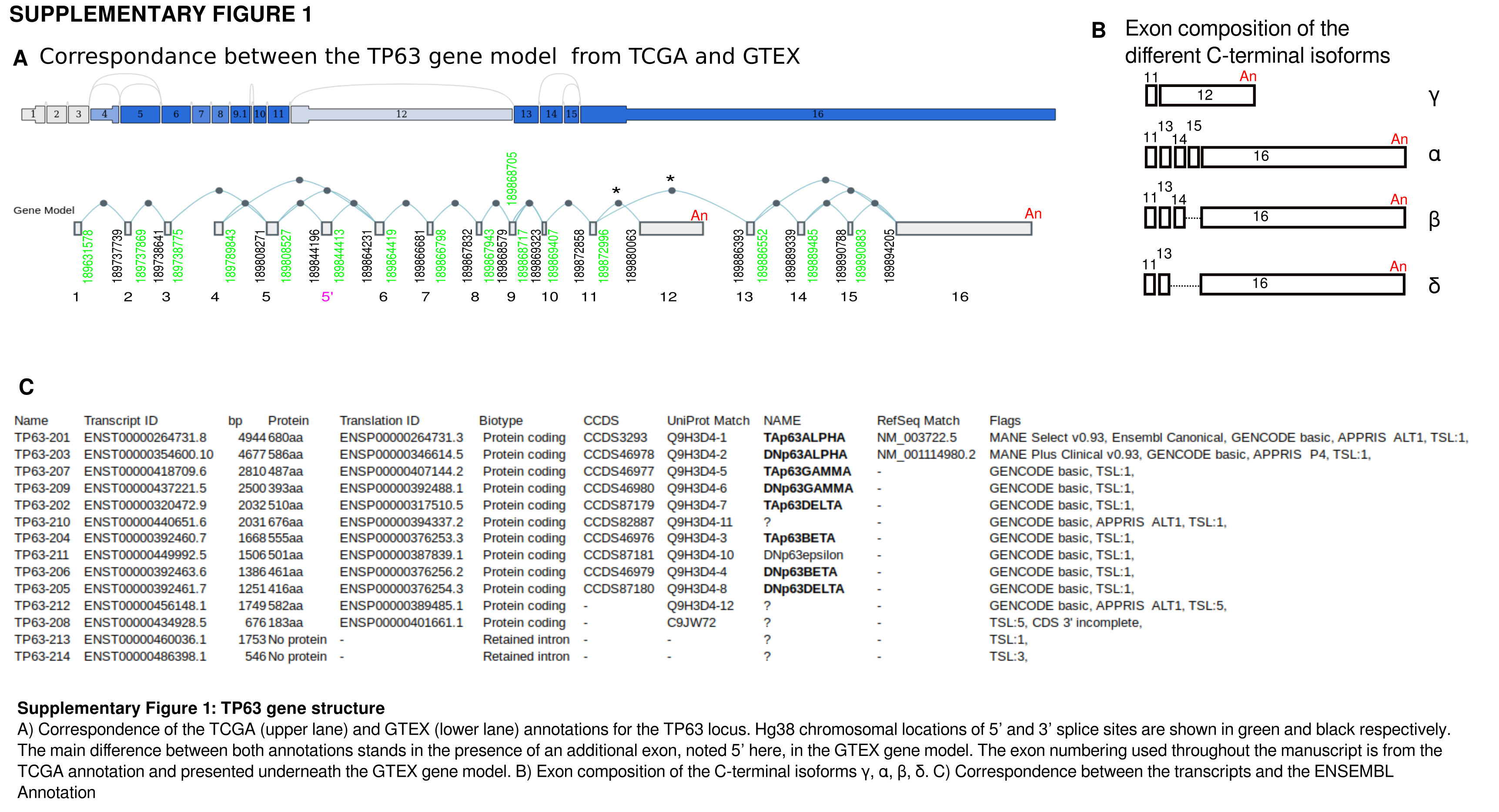

Supplement: Figure S1 — TP63 gene structure and annotation [file crc-22-0350-s05.png]

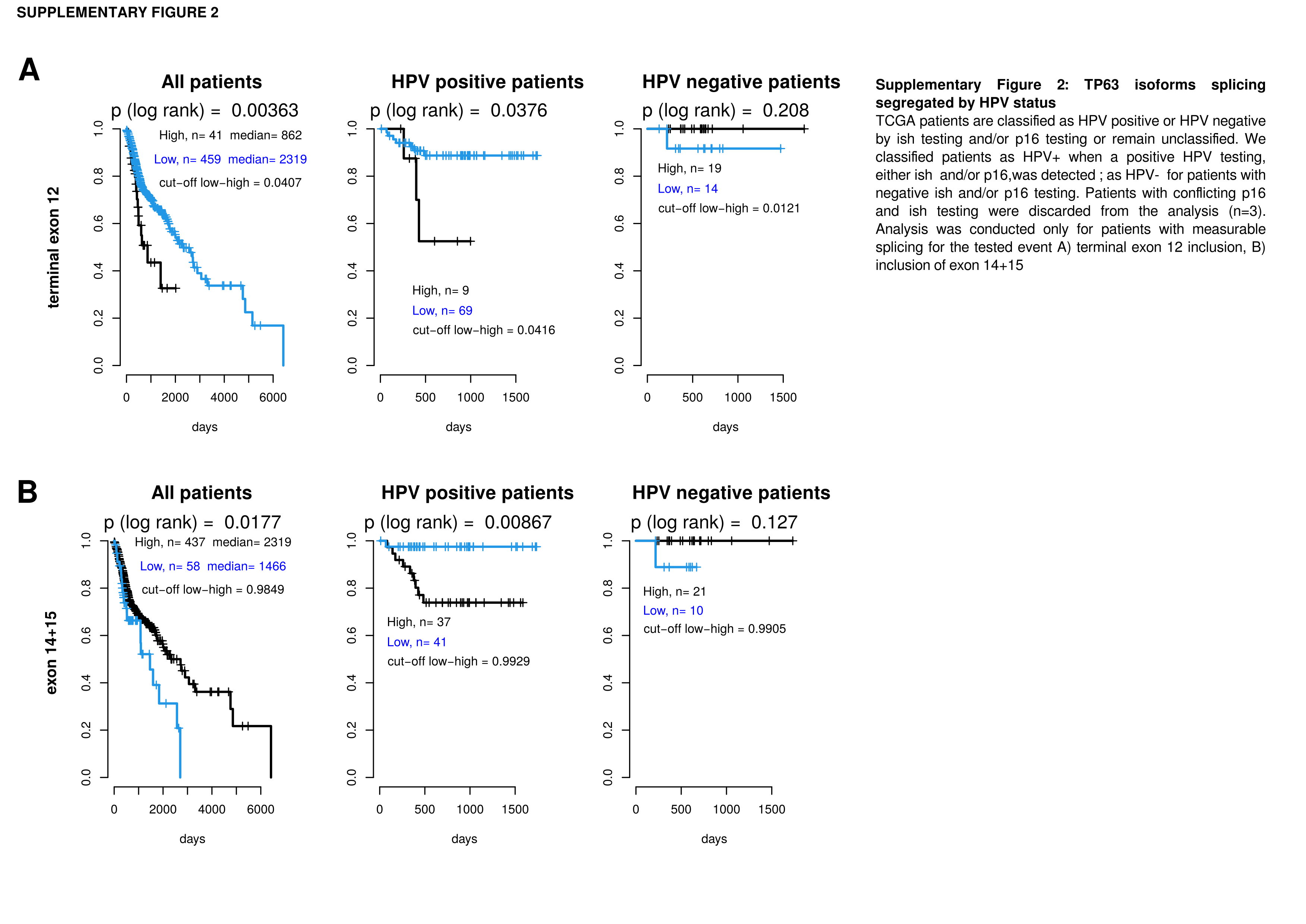

Supplement: Supplementary Figure 2 — TP63 isoforms splicing in patients segregated by HPV status [file crc-22-0350-s06.png]

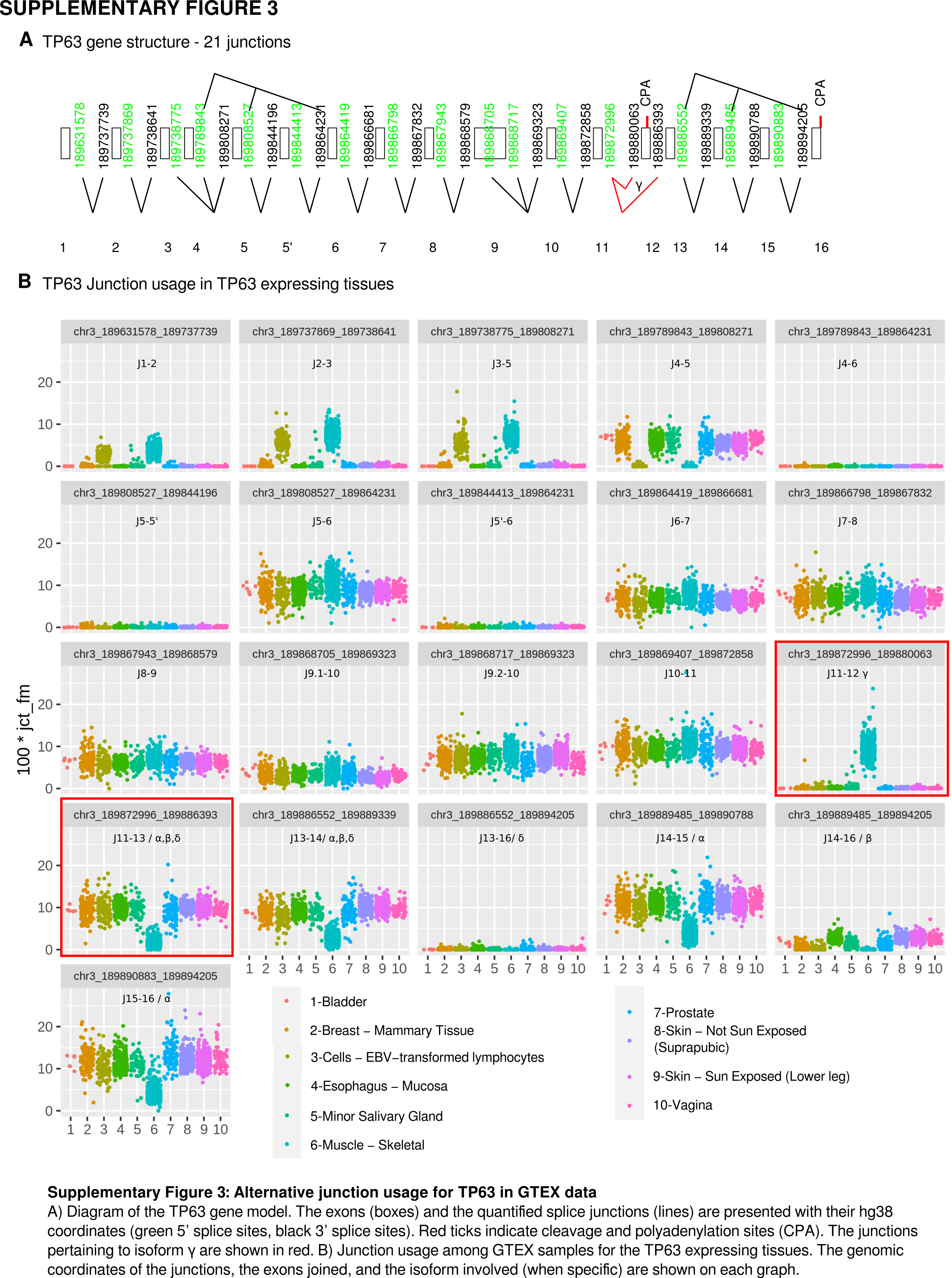

Supplement: Supplementary Figure 3 — Alternative junction usage for TP63 in GTEX data [file crc-22-0350-s07.png]

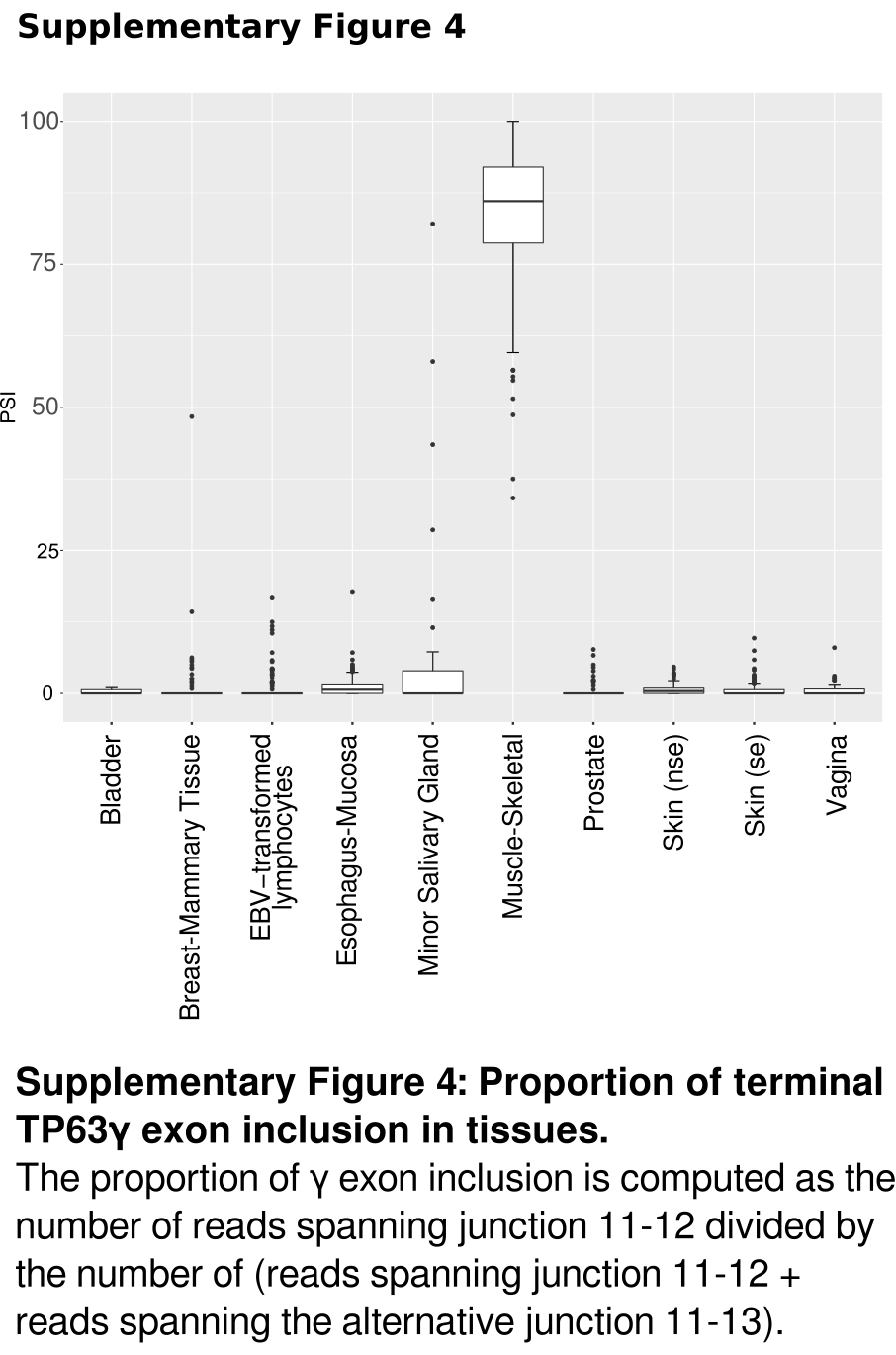

Supplement: Supplementary Figure 4 — Terminal TP63 gamma exon in tissues [file crc-22-0350-s08.png]

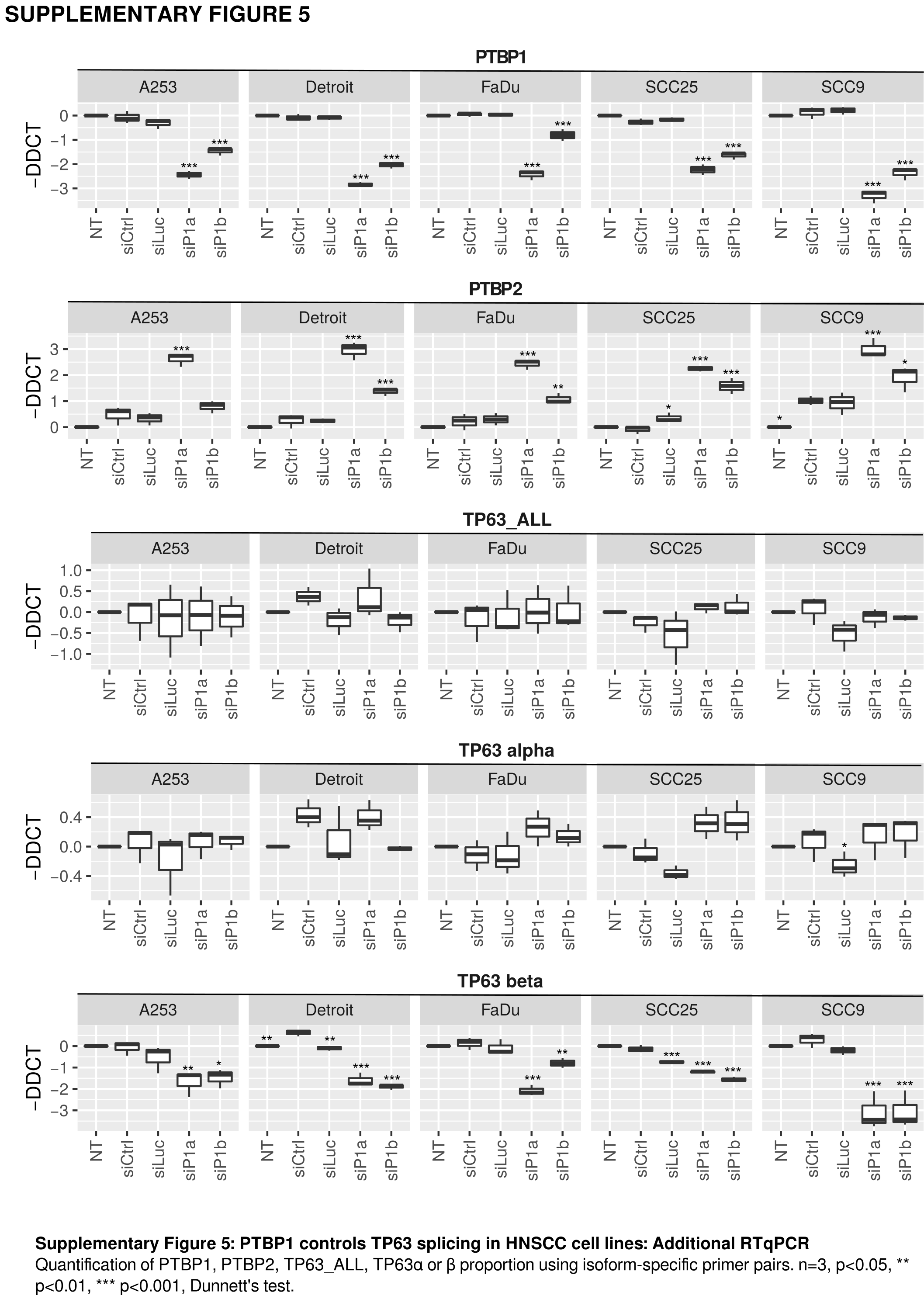

Supplement: Supplementary Figure 5 — PTBP1 controls TP63 splicing in HNSCC, additional RTqPCR [file crc-22-0350-s09.png]

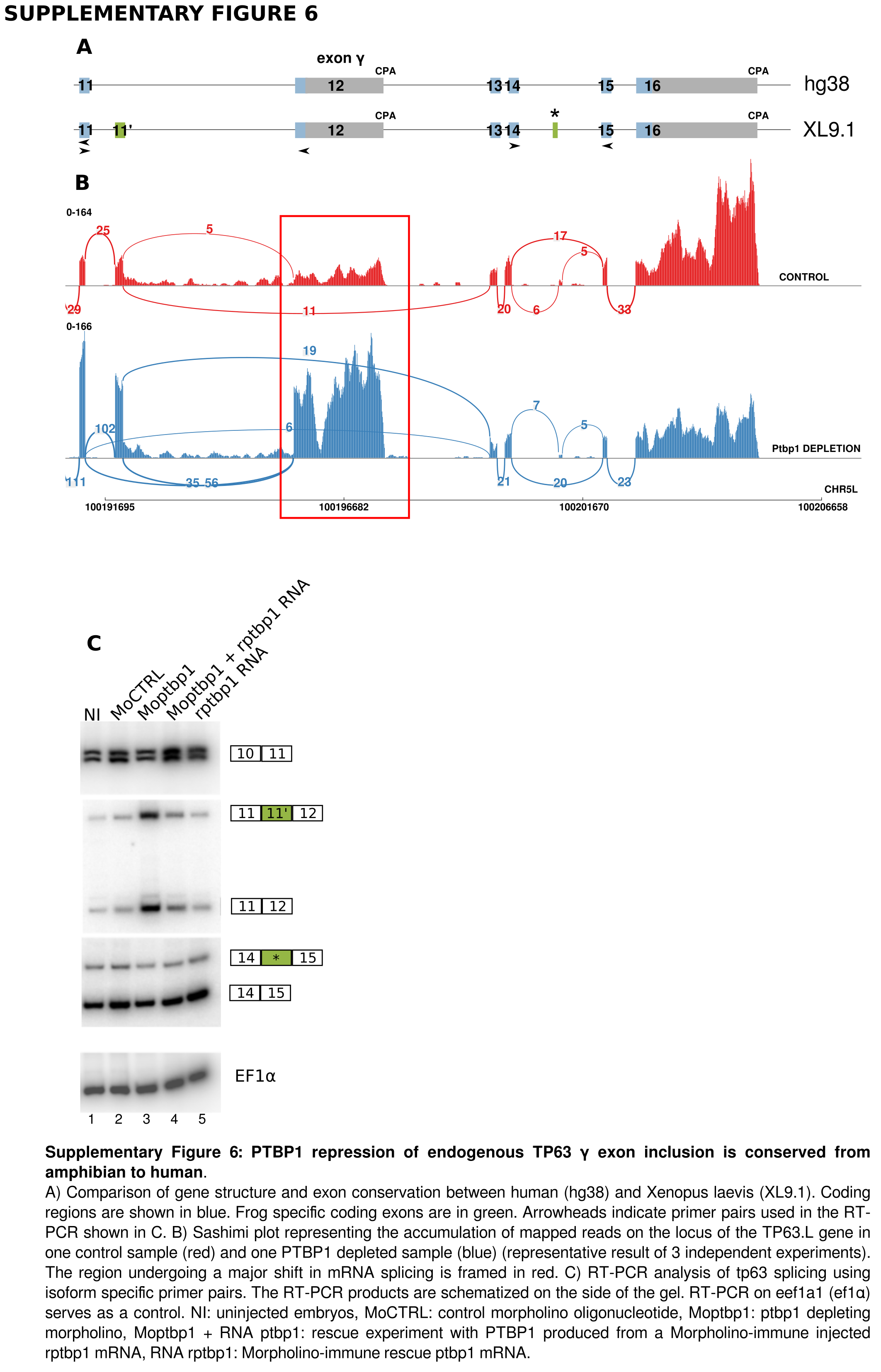

Supplement: Supplementary Figure 6 — Conservation of TP63 splicing regulation by PTBP1 in vertebrates [file crc-22-0350-s10.png]

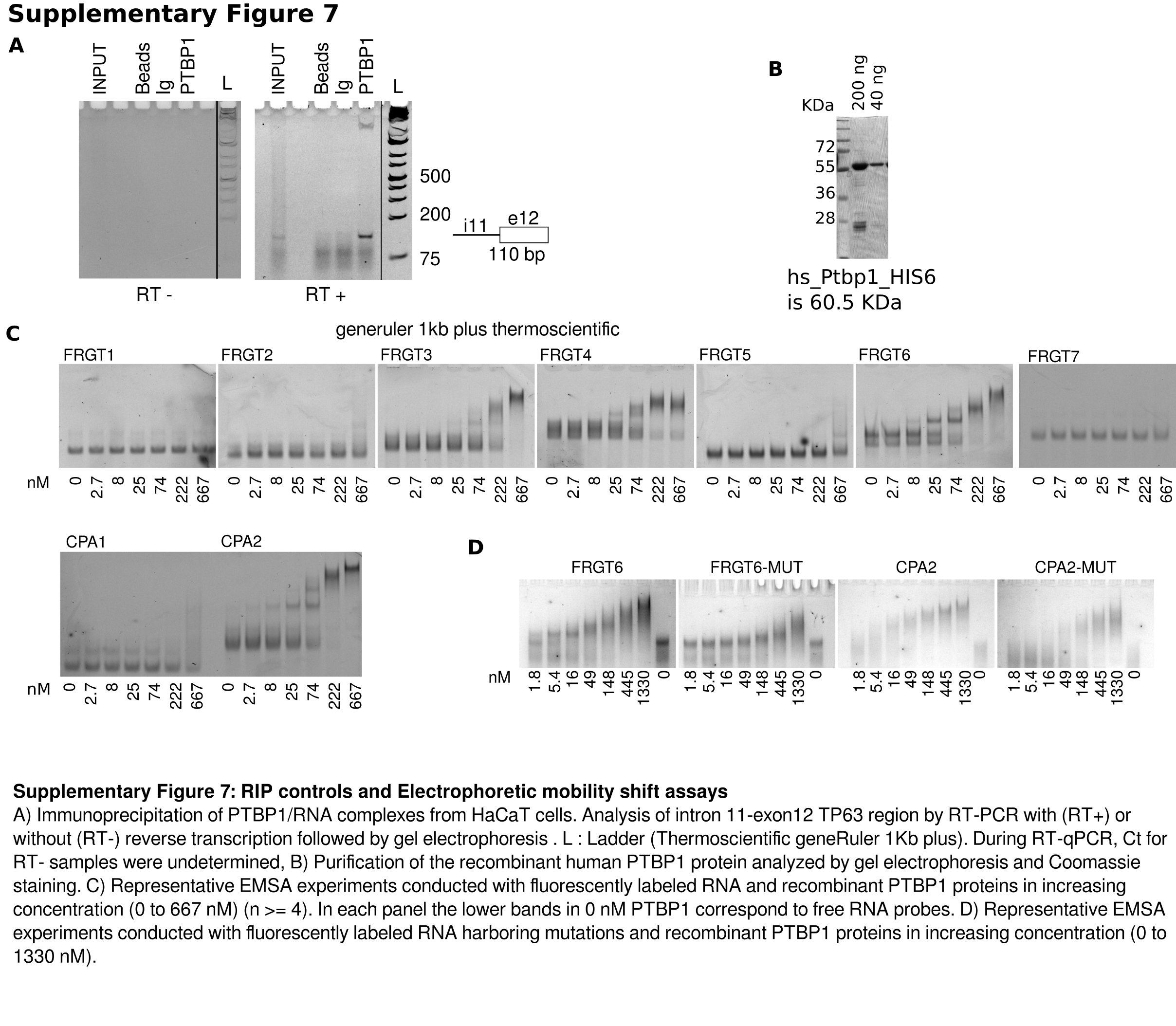

Supplement: Supplementary Figure 7 — RIP controls and EMSA [file crc-22-0350-s11.png]

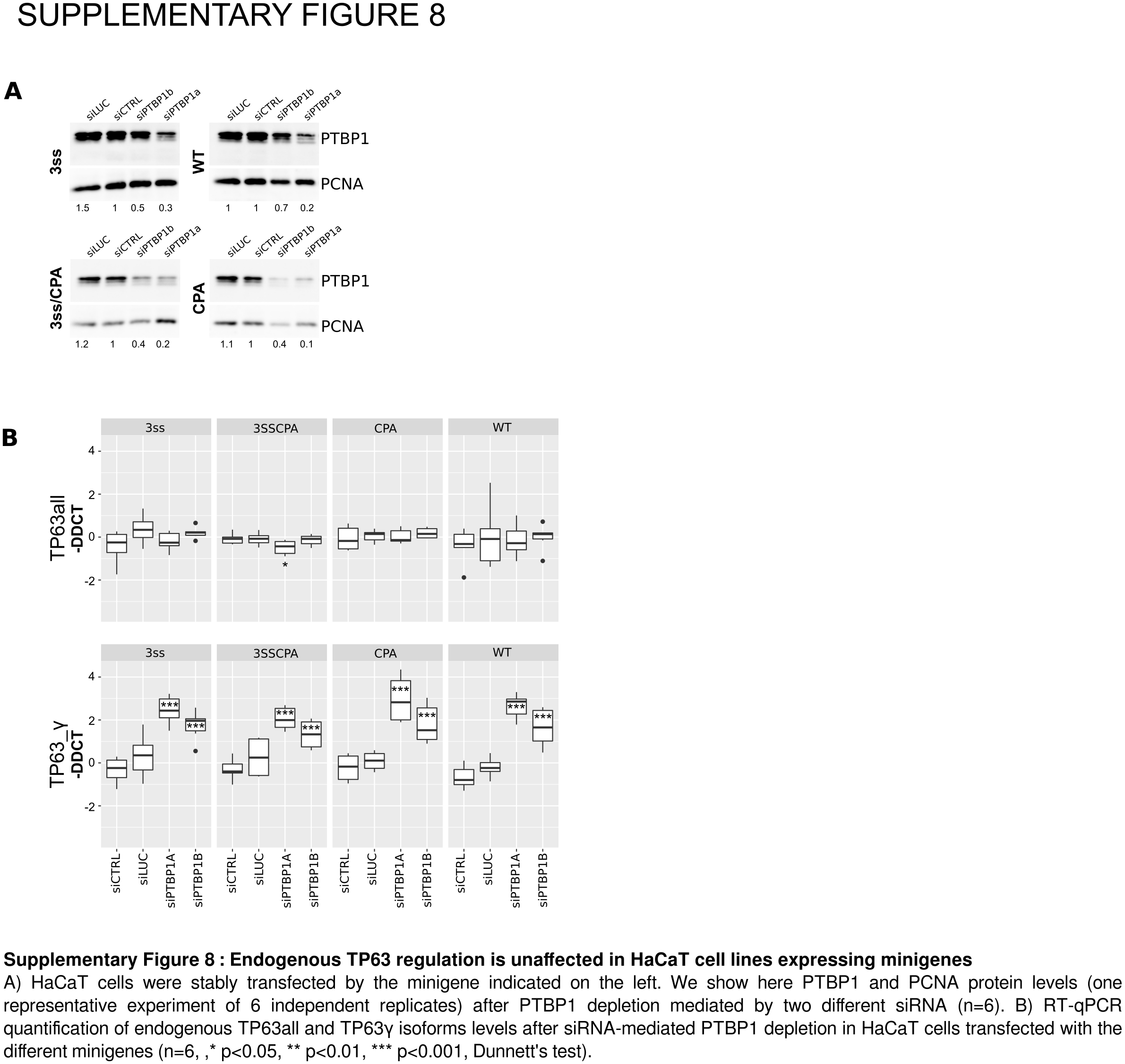

Supplement: Supplementary Figure 8 — endogenous TP63 regulation is unaffected in HaCaT cell lines expressing minigenes [file crc-22-0350-s12.png]
